# An Observer–Data-Path Decoupled Time-Domain ADC with Three-Stage Digital Background Calibration in 28 nm CMOS

**DOI:** 10.3390/mi17101110

**Published:** 2026-09-23

**Authors:** Chuan Qin, Bowen Liu, Runqiao Wang, Lecheng Li, Chaoyang Zhang

**Affiliations:** Faculty of Integrated Circuit, Xidian University, Xi’an 710071, China; 23211215034@stu.xidian.edu.cn (C.Q.); rqwang_6@stu.xidian.edu.cn (R.W.); lcli@stu.xidian.edu.cn (L.L.); zyzhang_0913@163.com (C.Z.)

**Keywords:** time-domain ADC, VTC, GRO-TDC, digital background calibration, observer–data-path decoupled, EWMA-LMS adaptation, nonlinearity calibration

## Abstract

This paper presents an observer–data-path decoupled time-domain ADC with three-stage digital background calibration, designed and comprehensively verified through post-layout simulations in 28 nm CMOS for highly integrated micro-systems. In scaled nodes, the voltage-to-time converter front-end exhibits large-signal nonlinearity, while the time-to-digital converter (TDC) resolution drifts with PVT; conventionally these two errors are strongly coupled in the time domain, making concurrent calibration difficult. To break this coupling, the proposed observer–data-path decoupled architecture confines a single-bit pseudo-random dither to an observer path for error extraction and reconstructs the signal in a dither-free data path, thereby separating the calibration loops without degrading the SNDR. Building on this decoupling, a constant time-offset injection logic eliminates the gated ring oscillator-TDC dead-zone, and the three-stage calibration compensates the ADC gain error induced by TDC-resolution drift through VTC-gain tuning, regulates the non-integer dither ratio through Bang-Bang control, and suppresses the residual even- and odd-order nonlinearity through a dither-modulated threshold-crossing algorithm with EWMA-filtered LMS polynomial adaptation. In post-layout simulation at 1 GS/s, the calibration lifts the near-Nyquist SFDR from 50.3 to 67.0 dBc and the SNDR to 46.0 dB, and improves the INL to −0.15/+0.70 LSB. The design consumes 2.98 mW under nominal conditions and 3.89 mW at the worst-power PVT corner, corresponding to nominal and worst-case Walden FoMs of 18.3 and 23.9 fJ/conversion-step, respectively, within a 0.0024-mm^2^ core.

## 1. Introduction

With the rapid proliferation of highly integrated micro-systems—such as micro-LiDAR for autonomous navigation, portable wideband sensor nodes for the IoT, and SoC communication interfaces—the demand for high-speed and power-efficient ADCs has grown dramatically. To pursue higher integration density and lower power consumption, these micro-systems are increasingly fabricated in advanced deep-submicron CMOS technologies. However, the continuous scaling of the supply voltage severely degrades the intrinsic gain of analog amplifiers and shrinks the available voltage headroom, pushing conventional voltage-domain ADCs against a fundamental dynamic-range bottleneck [1].

To overcome this barrier, time-domain ADCs (TD-ADCs) and voltage-controlled oscillator (VCO)-based ADCs have emerged as highly promising architectures [2,3,4,5]. By encoding the input voltage into a time interval or an oscillator phase prior to quantization, these architectures fully exploit the ultra-fast switching speed of scaled CMOS transistors, thereby exhibiting excellent process scalability and high energy efficiency that are particularly well suited to miniaturized electronics [3]. Although this same device-speed advantage has also enabled conventional architectures, such as single-channel SAR converters, to reach the gigahertz-rate regime [6], TD-ADCs and VCO-based modulators inherently bypass the voltage-headroom bottleneck by performing quantization directly in the time or phase domain, thereby achieving outstanding energy efficiency [4,5].

Despite these inherent advantages, realizing a high-performance GS/s TD-ADC in advanced nodes entails several critical challenges. First, the voltage-to-time converter (VTC)—the performance-defining front-end—suffers from severe nonlinearity under large input swings, owing to the finite output impedance of its current sources and to voltage-dependent parasitic capacitances [7,8]. Second, the time resolution *t_LSB_* of the time-to-digital converter (TDC) is highly sensitive to PVT variations; in densely integrated micro-systems, local thermal gradients can induce significant *t_LSB_* drift that translates into pronounced global gain errors [9]. Third, as the differential input approaches zero, the generated time pulse becomes too narrow to reliably trigger the subsequent TDC, giving rise to a severe quantization dead-zone and potential metastability [10].

To mitigate these non-idealities, digital background calibration is indispensable, and it has become a standard means of restoring linearity in high-resolution converters [11]. Conventional techniques typically inject a pseudo-random dither directly into the main data path to extract the error coefficients [12], and Least Mean Squares (LMS)-based adaptation driven by time-domain residues has been widely adopted in TDC- and PLL-related contexts [13]. The dither-injection approach, however, inevitably consumes a portion of the ADC dynamic range and degrades the SNR whenever the dither cannot be perfectly subtracted. Moreover, in high-speed TD-ADCs the *t_LSB_* drift and the VTC nonlinearity are deeply coupled in the time domain, so that conventional calibration loops tend to interfere with one another, making stable and fast convergence exceedingly difficult to attain at high speed and high resolution [14].

Fundamentally, the difficulty of convergence in conventional multi-parameter background calibrations arises from a “coupled oscillation” between analog tuning and digital weight updates. Let us denote the linear gain of the VTC as *K_VTC_* and its third-order nonlinearity coefficient as *α*_3_. In a shared-path architecture, the statistical expectation of the output code *D_out_*, which drives the gain tracking, is mathematically coupled with the uncalibrated nonlinearity:

(1) $$E\left[\left|D_{out}\right|\right]\approx \frac{K_{VTC}}{t_{LSB}}E\left[\left|V_{in}\right|\right]-\frac{\alpha_{3}}{t_{LSB}}E\left[{\left|V_{in}\right|}^{3}\right],$$

where *t_LSB_* is the time resolution. When a digital LMS loop updates the nonlinear compensation weight, it perturbs the data path and alters *E*[*|D_out_|*]. This forces the background loop to falsely adjust the analog VTC bias current to restore the gain *K_VTC_*. However, modifying the bias current physically alters the large-signal charging profile, thus changing the actual physical nonlinearity *α*_3_:

(2) $$\Delta \alpha_{3}\propto \Delta K_{VTC}\Rightarrow \frac{\partial \alpha_{3}}{\partial K_{VTC}}\neq 0$$

This creates an unstable cyclic dependency: the digital update perturbs the analog gain, which physically changes *α*_3_, thereby re-triggering another digital update. To strictly break this cycle and achieve robust convergence, an observer–data-path decoupled architecture is highly necessitated.

In this paper, a 1-GS/s 8-bit TD-ADC is designed and extensively verified through post-layout simulations with full parasitic extraction in a 28 nm CMOS process. The present work focuses on circuit-level and architectural verification; silicon measurement is not included, and all performance results are therefore explicitly reported as post-layout simulated results. To break the bottleneck of conventional coupled calibration, an observer–data-path decoupled architecture is introduced: errors are extracted in a dither-injected observer path while the signal is reconstructed in a dither-free data path, thereby decoupling the dither-based error-observation path from the clean data path and substantially reducing direct interference among the calibration loops without sacrificing the main-signal SNR, demonstrating a robust circuit-algorithm co-design paradigm. At the circuit level, a constant time-offset injection (CTOI) logic is incorporated to inherently eliminate the quantization dead-zone by shifting the zero-crossing point. Furthermore, a rigorous three-stage digital background calibration methodology is developed, which (i) compensates the ADC gain error caused by *t_LSB_* drift through VTC-gain tuning based on signal-swing statistics and a 7-bit monotonic current-tuning actuator, (ii) locks the dither ratio *Tdither*/*t_LSB_* through a Bang-Bang mixed-signal regulation loop, and (iii) suppresses the residual even- and odd-order nonlinearity through a dither-modulated threshold-crossing statistical algorithm that adaptively extracts second- and third-order polynomial coefficients via an exponentially weighted moving average (EWMA)-LMS engine.

## 2. System Architecture

The overall architecture of the proposed time-domain ADC is shown in Figure 1. Implemented in 28 nm CMOS, the ADC targets a sampling rate of 1 GS/s and a resolution of 8 bits for highly integrated micro-systems. Structurally, the system consists of three parts: an analog front-end, a time-domain quantization core, and a digital background calibration engine.

The differential inputs *VINP* and *VINN* are first sampled by a bootstrapped sampling switch to preserve good sampling linearity at high speed, and are then fed to a VTC, which translates the input voltage difference into a pair of differential time pulses whose width difference is proportional to the input voltage, thereby completing the mapping from the voltage domain to the time domain. The VTC is powered by a current-controllable low-dropout regulator (LDO); by adjusting its output current, the conversion slope of the VTC can be tuned, providing the physical means for compensating the ADC gain error caused by *t_LSB_* drift. To eliminate the quantization dead-zone for small input signals, a CTOI logic introduces a fixed time offset during pulse generation, shifting the quantization operating point away from the zero-differential point. Following the CTOI, the time-domain signal splits into two parallel paths: the main data path, which preserves the original time pulses (*EN*), and the observer path, where a pseudo-random time offset (*Tdither*) is conditionally injected to generate the dithered pulses (*END*). Subsequently, these two sets of time pulses are quantized by a gated ring oscillator (GRO)-TDC into 7-bit raw digital codes. To ensure independent quantization for each sample, the GRO-TDC employs a per-conversion reset mechanism that clears the residual phase before every conversion, thereby preventing quantization error accumulation and maintaining inter-conversion consistency.

In the digital domain, the system is partitioned into two mutually decoupled branches: a data path and an observer path. In every conversion the GRO-TDC provides two successive snapshots of the same GRO state within each conversion, the dither-free code *D_EN* routed to the data path and the probed code *D_END* routed to the observer path. Whether *D_END* differs from *D_EN* is governed by a single-bit pseudo-random sequence *d*[*n*] ∈ {0, 1}, generated by an on-chip pseudo-random binary sequence (PRBS) generator: only in the cycles with *d*[*n*] = 1 does the observer path inject a fixed, non-integer time-offset probe *Tdither*. The data path applies nonlinearity compensation to the clean code *D_EN* and directly forms the ADC output *D_out_*, and it also provides the code-magnitude statistics used by the Stage-1 gain calibration, whereas the observer code *D_END* is used to regulate the dither ratio and extract nonlinear-error information; these coefficients are then fed back to the data path through a three-stage digital background calibration scheme. Because the data path never carries the probe, this decoupled design enables robust error extraction and compensation without sacrificing the dynamic range or the SNR of the main signal.

Centered on this architecture, the core innovations of this work include the observer–data-path decoupled architecture, the CTOI dead-zone elimination logic, and the three-stage digital background calibration method. Their circuit implementations and algorithmic principles are elaborated in Section 3, in which the analog and time-domain circuits are described in Section 3.1, Section 3.2 and Section 3.3 and the digital background-calibration algorithm is developed in Section 3.4.

## 3. Circuit Design and Implementation

This section presents the circuit implementation following the signal flow from the VTC front end to the GRO-TDC and digital calibration engine. Section 3.1, Section 3.2 and Section 3.3 describe the analog and time-domain circuits, followed by the three-stage background calibration in Section 3.4.

### 3.1. VTC Front-End

#### 3.1.1. Bootstrapped Sampling and VTC Conversion Principle

The core circuit of the VTC front-end is shown in Figure 2.

In the analog front-end, to mitigate charge injection and clock feedthrough effects, a slightly advanced clock phase, denoted as *CLKSa*, is employed to turn off the bottom-plate switch slightly before *CLKS* goes low. During the hold phase, the complementary clock, denoted as $\bar{CLKS}$, is activated. The differential inputs *VINP* and *VINN* are sampled by the sampling switches after the input buffer: during the sampling phase (*CLKS* high), the sampling capacitors are charged to track the inputs, yielding the sampled node voltages *Vsp* and *Vsn*. To suppress the harmonic distortion introduced by a signal-dependent on-resistance at the high sampling rate of 1 GS/s, the sampling switches are driven by the bootstrapped voltages *Vboostp* and *Vboostn*, generated by the circuit shown in Figure 3. Based on a charge pump and a source follower, this bootstrapping circuit superimposes a constant boosted voltage onto the input and applies it to the gate of the sampling transistor during the sampling phase, keeping the gate-source voltage *V*_GS_ approximately constant over the entire input swing [7,15].

In the gate-bootstrapping circuit, M3 operates as a source follower to output the boosted voltage, and its source node corresponds to Vs. As shown in Figure 3, node C is the internal storage node of the charge pump. When the circuit enters the hold phase, node C suffers from a slow leakage, and its voltage disturbance couples to the source node *Vs* through the overlap-fringing capacitance *C_of_* of M3, thereby perturbing the sampled voltage and degrading the hold accuracy. To eliminate this coupling path, a switch transistor M4 is introduced below node C: during the hold phase ($\bar{CLKS}$ active), M4 turns on and rapidly clamps node C to ground, keeping its voltage stable and thus cutting off the coupling from the leakage of node C to *Vs* through *C_of_*, which guarantees the stability of the sampled voltage and the sampling accuracy during the hold phase.

The conversion phase follows the sampling. As shown in Figure 2, the VTC adopts a current-charging topology: a charging current *I_S_* charges the capacitance at the output nodes, so that the node voltages *Vsampp* and *Vsampn* rise continuously from their sampled values; once a node voltage reaches the trip threshold *V_TH_* of the time comparator, the comparator toggles and produces a time-pulse edge, outputting *TINP* and *TINN*. Since the charging starting points of the positive and negative branches are set by the differential input, the two branches reach VTH at different instants, thereby encoding the differential input voltage into a differential time difference. The conceptual conversion waveforms are illustrated in Figure 4a, and the corresponding post-layout simulation curves of the VTC front-end are presented in Figure 4b, which verify the expected charging behavior and time-pulse generation.

Ideally, if the charge current *Is* were constant, the charge-node voltage would rise linearly with time:

(3) $$Vsamp\left(t\right)=Vsamp\left(0\right)+\frac{I_{S}}{C_{S}}t,$$

where *C_S_* is the equivalent capacitance at the charging node. Setting *Vsamp* to *V_TH_* yields the charge time of each branch, and their difference gives the output differential time difference. For a differential input *VINP* − *VINN* = Δ*V_in_*, this leads to

(4) $$\Delta t=TINN-TINP=\frac{C_{S}}{I_{S}}\Delta V_{in}$$

Accordingly, the voltage-to-time conversion gain of the VTC is *K_VTC_* = *C_S_/I_S_*. For the cascaded VTC–TDC conversion, the digital output code is approximately proportional to

(5) $$D_{\mathrm{out}}\propto \frac{\Delta T}{t_{LSB}}=\frac{K_{VTC}}{t_{LSB}}\Delta V_{in}$$

where *t_LSB_* denotes the physical time resolution of the GRO-TDC. Therefore, a PVT-induced variation of $t_{LSB}$ appears as an overall voltage-to-code gain error. Importantly, the proposed Stage-1 calibration does not physically tune the GRO-TDC delay or *t_LSB_*. Instead, it adjusts the VTC charging current *I_S_*, and hence *K_VTC_*, to compensate for the conversion-gain variation caused by the GRO-TDC *t_LSB_* drift. This complementary VTC-gain tuning constitutes the physical basis of the 7-bit LDO-assisted Stage-1 gain calibration.

Equations (3) and (4), however, implicitly assume a constant *I_S_*. In practice, the current source providing *I_S_* has a finite output impedance *r_o_*, so that its charging current varies with the rising node voltage *Vsamp*, rendering the charging process not strictly linear and thus exhibiting a certain nonlinearity. Taking this effect into account, the single-ended charge current can be expanded to first order as

(6) $$I\left(Vsamp\right)\approx I_{S}-\frac{Vsamp}{r_{o}},$$

where the term *Vsamp*/*r_o_* represents the voltage-dependent nonlinear current introduced by the finite output impedance, causing the single-ended charge time to deviate from the ideal linear relationship and introducing an error term *δ*(*Vsamp*). Nevertheless, owing to the fully differential structure adopted in this design, the positive and negative branches feature perfectly symmetric charging paths and identical current-source characteristics, so that the nonlinear error terms they introduce are equal. When the two branches are subtracted to extract the differential time difference, this nonlinear component is canceled to first order as a common-mode term, i.e.,

(7) $$\Delta t=\left[t_{n}+\delta \left(V_{sn}\right)\right]-\left[t_{p}+\delta \left(V_{sp}\right)\right]\approx \frac{C_{S}}{I_{S}}\Delta V_{in}$$

Equation (7) indicates that the nonlinear current arising from the finite output impedance appears, to first order, as a common-mode (even-order) term under the fully differential architecture and is therefore effectively canceled upon differential subtraction; it does not degrade the differential conversion linearity. This property allows the proposed VTC front-end to maintain good first-order linearity even under large input swings. However, the differential subtraction cancels only the even-order component. Under large input swings the voltage-dependent charging current also produces a higher-order, differential-mode nonlinearity—dominated by the odd-order (HD3) slope compression—which cannot be removed by differential cancelation and instead manifests as the S-shaped integral-nonlinearity (INL) profile observed in Section 4. This residual differential-mode nonlinearity is the error component that the dither-modulated threshold-crossing adaptive calibration of Section 3.4.2 is specifically designed to extract and compensate.

#### 3.1.2. 7-Bit Controllable LDO and VTC Gain Calibration

The charging current *I_S_* of the VTC sets its conversion gain *K_VTC_* = *C_S_*/*I_S_*. Since the overall voltage-to-code gain is proportional to *K_VTC_*/*t_LSB_*, the VTC gain provides a convenient analog degree of freedom for compensating the ADC gain error caused by PVT-dependent GRO-TDC resolution. Accordingly, a 7-bit current-controllable LDO generates a tunable bias current that is mirrored into *I_S_*; the circuit is shown in Figure 5. It should be emphasized that this actuator modifies *K_VTC_*, rather than the physical *t_LSB_* of the GRO-TDC.

As shown in Figure 5, the LDO uses a bandgap reference and forms a closed loop through an error amplifier and a current-source array, generating the calibration current *I*dis at the output stage M3 and M4, which is made digitally tunable by the VTC current calibration unit. Transistors M1 and M2 in the VTC (see Figure 2) share the bias voltages VB1 and VB2 with the output-stage transistors M3 and M4 of the LDO, forming a current mirror, so the actual charging current is proportional to the calibration current:

(8) $$Is=N\cdot I\_dis=N\cdot \left(I\_base+ISW\langle 6:0\rangle \cdot I\_unit\right),$$

where *N* is the mirror ratio, *I_base* is a fixed reference current, Iunit is the unit step current of the LDO, and *ISW*⟨6:0⟩ is the 7-bit binary-weighted control word, with a power-on default code of the mid-scale value 100_0000 that reserves a bidirectional tuning range. Since *K_VTC_* = *C_S_*/*Is* and *Is* is mirrored from *Idis* by the ratio *N* (Equation (8)), tuning *ISW*⟨6:0⟩ monotonically adjusts the VTC conversion gain. This tuning range is used to counteract the overall ADC gain variation associated with the PVT-dependent *t_LSB_* of the GRO-TDC. Figure 6 illustrates the corresponding conceptual relationship between the VTC charging current and *K_VTC_*. As expected from *K_VTC_* = *C_S_*/*Is*, increasing the charging current reduces the voltage-to-time conversion gain, providing a monotonic actuator for compensating the overall ADC gain variation.

It should be distinguished that only the first-stage gain calibration physically modifies the VTC conversion slope by tuning *Is*. The subsequent *Tdither*/*t_LSB_* regulation is applied after the VTC in the observer path, while the polynomial correction is performed digitally; therefore, Stages 2 and 3 do not perturb the physical VTC characteristic or the clean EN path.

### 3.2. Time-Shaping Circuit

After the VTC, the differential input voltage is encoded into a pair of time pulses *TINP* and *TINN*, whose phase difference Δ*T* = *t*(*TINP*) − *t*(*TINN*) carries the input information. The time-shaping circuit is located between the VTC and the GRO-TDC as the intermediate stage of the signal chain: the time pulses produced by the VTC are first processed by the time-shaping circuit, the resulting enable pulses are then quantized by the GRO-TDC, and the quantization result is finally processed by the three-stage digital background calibration. The circuit performs two functions: deciding the sign bit with a time comparator, and generating the enable pulses *EN* and *END* for the quantizer with a dither-injection pulse generator (DIPG). The DIPG integrates two parts, CTOI and *Tdither* injection, which provide dead-zone compensation and the dither injection for nonlinearity calibration, respectively.

The time comparator decides which of *TINP* and *TINN* arrives first and outputs the most significant bit (sign bit) *DMSB* of the quantization result. Since dither injection takes place later in the DIPG, the sign decision is based solely on the original time difference Δ*T* and is therefore independent of dither. To achieve low-offset operation under the scaled supply, the time comparator adopts a double-tail dynamic topology [16]. It is worth noting that the Monte-Carlo simulation in Figure 7b is an end-to-end front-end evaluation including the current-controllable LDO-biased VTC and the time comparator. The LDO is included in the simulation because its bias-current variation directly affects the VTC charging slope. Global and local device variations are considered together with the evaluated supply and temperature conditions. The Monte-Carlo analysis was repeated across VDD = 0.95/1.0/1.1 V and T = −40/65/125 °C. A total of 200 Monte-Carlo samples were used for the reported statistical evaluation; the extracted one-sigma spread had already become essentially stable after approximately 100 samples. By applying a slow ramp voltage signal to the VTC and monitoring the time comparator’s final decision, the overall equivalent time offset is extracted. Thus, the simulated offset intrinsically includes the static mismatch and offset contributions from the VTC. The comparator circuit and its Monte Carlo offset simulation are shown in Figure 7; the simulated offset mean and standard deviation indicate a three-sigma value below ½ LSB, ensuring a reliable sign decision near the zero crossing.

The subsequent stage uses the GRO-TDC for time quantization. Owing to gate delay and the GRO start-up time, a portion of the effective enable time is consumed in every quantization cycle. Near Δ*T* ≈ 0 (the signal zero crossing), the pulse becomes too narrow to be reliably quantized, creating a quantization dead zone that degrades ADC linearity. This issue is common to gated-ring-oscillator-based TDCs [10,14].

The DIPG employs the CTOI logic to eliminate the dead zone; its pulse-generation logic is shown in Figure 8. *TINN* is delayed through two branches *T_d_*_1_ and *T_d_*_2_ and XOR-combined into *TN*1, while *TINP* is delayed through the cross-connected *T_d_*_2_ and *T_d_*_1_ branches and XOR-combined into *TP*1; both pulses have a constant width:

(9) $$Tcom=\left|T_{d1}-T_{d2}\right|$$

*TN*1 and *TP*1 are then OR-combined into the main enable pulse *EN*:

(10) EN=TN1+TP1,

(11) $$W_{EN}=\left|\Delta T\right|+Tcom,$$

where *W_EN_* is the pulse width of *EN*. As shown in (11), the constant *Tcom* shifts the zero crossing along the time axis so that *W*EN always satisfies *W_EN_* ≥ *Tcom*, physically moving the operating point away from the GRO-TDC dead zone. *Tcom* appears in the digital output merely as a fixed DC offset, which can be directly removed in the digital domain without reducing the effective quantization range.

To correct the quantization nonlinearity introduced by the VTC and the GRO-TDC, the DIPG generates a second enable pulse *END* with injected dither in addition to *EN*. As shown in Figure 8a, *EN* is passed through a controllable delay chain (Delay = *Tdither*); the delayed pulse is gated by the per-cycle dither bit *d*[*n*] and then OR-combined with *EN* itself to form *END*:

(12) $$END\left[n\right]=EN\left[N\right]OR\left(d\left[n\right]\cdot Delay\left(EN\left[n\right],Tdither\right)\right)$$

Thus *END* and *EN* always share aligned rising edges, while their falling edges differ by *Tdither* only in the dithered cycles:

(13) $$t\_fall\left(END\left[n\right]\right)-t\_fall\left(EN\left(n\right)\right)=d\left[n\right]\cdot Tdither,d\left[n\right]\in \left\{0,1\right\}$$

The dither in this design is single-bit. In every conversion the GRO-TDC quantizes both *EN* and *END*, but whether *END* actually carries the extra delay is decided by a per-cycle pseudo-random dither bit *d*[*n*] ∈ {0, 1} produced by an on-chip PRBS generator: when *d*[*n*] = 1 a fixed delay *Tdither* is injected, so that *END* lags *EN* by *Tdither* at the falling edge; when *d*[*n*] = 0 no delay is injected and *END* coincides with *EN*. The injected amount is thus deterministic and fixed (a constant *Tdither*), while its presence is randomized by the single-bit sequence *d*[*n*]. The dither-injection unit forms the *Tdither* delay chain from multiple delay cells, whose individual delays are tuned by the differential control signals *ditherp* and *dithern*; their role is not to vary *Tdither* over time, but to stabilize the ratio *Tdither*/*t_LSB_* at a constant, non-integer value. This non-integer constraint is the key to exposing nonlinearity: if *Tdither*/*t_LSB_* were an integer, an injected sample would correspond merely to an integer-LSB shift in the digital domain, unable to cross nonlinear break points and therefore unable to reveal nonlinearity. With a non-integer ratio, the dithered (*d*[*n*] = 1) samples experience a different local gain from the non-dithered (*d*[*n*] = 0) ones at the same input level, and the pseudo-random switching decorrelates the probe from the input signal, thereby revealing the slope compression in the digital domain while providing the excitation required for the background calibration convergence [12]. A conceptually similar single-bit dithering principle has also been successfully exploited for timing-skew calibration in high-speed time-interleaved converters [17].

Both *D_EN* and *D_END* are obtained from the same physical GRO within a single conversion. Their rising edges are aligned, while the two falling edges define two successive observation instants of the same running GRO state. The detailed snapshot operation is described in Section 3.3. The resulting difference provides the digital-domain observation of the injected probe:

(14) $$D\_dither\left[n\right]=D\_END\left[n\right]-D\_EN\left[n\right]=d\left[n\right]\cdot \Delta D\left(D\_EN\left[n\right]\right),$$

where Δ*D*(*D_EN*) denotes the actual code increment produced by the fixed *Tdither* at the input code *D_EN*. For an ideally linear VTC this increment equals the nominal value *D_target* at any level; under slope compression it becomes smaller than *D_target*, which is the signature exploited by the nonlinearity calibration in Section 3.4.2.

It is important to emphasize that the *Tdither* probe is injected in the pure time domain (via the DIPG) strictly after the VTC front-end. Consequently, the intrinsic large-signal nonlinearity of the VTC is shared as a common-mode baseline by both the data path (*EN*) and the observer path (*END*), ensuring that the dither injection does not physically perturb the VTC transfer characteristics. Furthermore, while the analog delay cells in Figure 8b inherently exhibit a nonlinear delay-to-control-voltage transfer curve, this nonlinearity does not degrade the calibration accuracy. The background loop regulates *Tdither* to a single, static target (e.g., *D_target* = 8 LSB) rather than sweeping it across a wide range. For such single-point closed-loop regulation, only the tuning monotonicity of the delay chain is required, rendering its tuning nonlinearity irrelevant to the steady-state extraction of the VTC error.

It is worth noting that this design does not require subtracting the dither from the output. Since the probe is applied only to the *END* path and never to the main output *D_EN*, the difference *D_END* − *D_EN* in the dithered cycles directly provides an error observation of the quantization nonlinearity. Based on this observation, the sign-based Bang-Bang and EWMA-LMS algorithms in Section 3.4 perform background gain and nonlinearity calibration respectively on top of *D_EN*; because *D_EN* itself contains no injected dither, no dither component needs to be removed from the output. This is consistent with the established method of exposing nonlinearity with a single-bit dither and performing background calibration at the injection point [18].

In summary, the time-shaping circuit provides a reliable, dither-independent sign bit through the time comparator, eliminates the GRO-TDC quantization dead zone with a constant *Tcom* via CTOI, and supplies the observation for the nonlinearity background calibration through a non-integer *Tdither* injection with a stabilized ratio—together ensuring the linearity and robustness of the time-domain quantization.

### 3.3. GRO-TDC

The time quantization in this design is performed by a TDC based on a GRO. The GRO-TDC starts the ring oscillator while the enable pulse is high, freezes its phase when the pulse goes low, and uses both the number of oscillation cycles and the final phase state as the time-quantization result. Only one physical GRO is used to generate both *D_EN* and *D_END*. A duplicated GRO is deliberately avoided because relative oscillator mismatch would directly contaminate the dither observation while also increasing power and area. At the system level, the shared GRO is enabled by *END*, which is never shorter than *EN*. When *d*[*n*] = 0, *EN* and *END* terminate together and the two codes are identical. When *d*[*n*] = 1, *EN* terminates first while *END* remains asserted, so the same GRO continues oscillating during the additional *Tdither* interval. The running phase state is first captured at the falling edge of *EN* to form *D_EN*; the GRO then continues running until the falling edge of *END*, after which the final counter/phase state is captured to form *D_END*. Therefore, the two codes are two snapshots of the same GRO trajectory rather than outputs of two separate oscillators.

The two snapshots have different timing requirements. *D_EN* is captured while *END* is still high and the GRO remains active, so this intermediate snapshot does not depend on post-stop phase retention. By contrast, *D_END* is captured after the GRO is stopped and therefore uses the phase-tracking *CLKF* mechanism to ensure that the sampling event falls within the valid *T*_1_–*T*_2_ retention window.

The retained phase state during each enable interval allows the final oscillator phase to be used for fine time quantization, while the resettable inverters return the loop to a defined initial phase before every conversion, preventing residual phase from accumulating across samples; this combination makes it a common structure for high-resolution time quantization [10]. The circuit implementation of the GRO-TDC is shown in Figure 9, where (a) is the overall GRO circuit and (b) is the interpolation cell. The interpolation cell inserts an intermediate time signal between two adjacent time signals, effectively subdividing the quantization step below a single delay stage and thereby improving the time resolution without shortening the inverter delay. The basic cells forming the loop are shown in Figure 10: (a) is the GRO oscillation cell and (b) is the resettable inverter, whose resettable structure returns the loop to a defined initial phase before each quantization, avoiding phase-accumulation error.

For clarity, the label *EN* in the GRO-cell schematics of Figure 9 and Figure 10 denotes the local oscillator-enable terminal; in the complete ADC, this terminal is driven by the system-level *END* signal.

The performance of the GRO-TDC depends largely on the accurate generation of the sampling clock, i.e., the GRO phase state must be latched at the proper instant. The conventional fixed-delay sampling-clock scheme suffers from three main technical bottlenecks.

First, PVT variations cause fluctuations in the GRO oscillation period, so a fixed-delay sampling clock cannot accurately track the actual instant at which the GRO stops oscillating, leading to sampling-phase mismatch;

Second, if sampling occurs before the GRO has fully settled, the loop is still in a transient state and is prone to metastability, causing quantization errors;

Third, the GRO phase information is stored as charge on parasitic capacitance; if sampling occurs too late, charge leakage shifts the stored phase and severely degrades system linearity.

The sampling must therefore fall within a finite valid window: as shown in Figure 11, it must be completed within the interval T1 to T2, after the GRO has stopped oscillating but before significant charge leakage occurs.

To always hit this window under PVT variations, this design adopts a phase-tracking sampling-clock generation circuit, shown in Figure 12.

The input time signals *TINN* and *TINP* are fed, on one path, into a DIPG (with delay *t*3) to produce the enable signal *END*, where *t*3 is defined as the delay from the earlier-arriving rising edge of *TINN* and *TINP* to the rising edge of *END*; on the other path, they are fed into an AND gate that selects the later-arriving rising edge, so that the edge of this branch adapts to the actual GRO stop instant. The AND-gate output is delayed by *t*1 to obtain *T*1, and *T*1 is further delayed by *t*2 to obtain *T*2; *T*1 and *T*2 are combined by a NAND gate to generate the sampling clock *CLKF*. It should be explicitly noted that *CLKF* is an internally generated, phase-tracking sampling clock dedicated solely to latching the GRO-TDC state. It is entirely distinct from the front-end analog sampling clock *CLKS*. From the NAND logic, *CLKF* produces a narrow low-level pulse during the interval in which *T*1 and *T*2 are simultaneously high, and the instant corresponding to this pulse constitutes the valid *T*1–*T*2 sampling window. The delay *t*1 is designed to be slightly larger than *t*3, ensuring that this sampling pulse appears only after the GRO has completely stopped oscillating, while the delay *t*2 sets the width of the sampling window. Since the entire clock chain is derived from the GRO’s own input edges, its triggering instant drifts in step with the GRO stop instant, allowing it to continuously track the stop instant under PVT variations while avoiding metastability and charge-leakage effects.

More importantly, the relative timing is enforced by the same-edge-derived delay relation rather than by two independent clocks. The design condition t1 > t3 guarantees that the CLKF sampling event is generated only after the GRO stop condition has been established. This ordering was maintained over the simulated PVT corners. On the late-sampling side, the charge-retention time of the frozen GRO nodes is substantially longer than the logic-delay difference used to generate CLKF; therefore, t2 can be selected conservatively so that CLKF remains inside the valid retention window even at the slowest corner.

In summary, the GRO-TDC in this section subdivides the time resolution below a single delay stage through the interpolation cell, and adaptively locks the sampling instant within the valid post-oscillation window through the phase-tracking sampling clock, thereby ensuring the accuracy and linearity of the time quantization under PVT variations.

### 3.4. Digital Background Calibration

#### 3.4.1. Gain and Dither-Ratio Calibration

The physical time resolution *t_LSB_* of the GRO-TDC is determined by the interpolated ring-oscillator delay and is therefore sensitive to PVT variations. As shown in Figure 13a, *t_LSB_* varies from 3.08 ps to 4.77 ps across the evaluated PVT conditions, which directly produces an overall ADC gain variation. Stage 1 does not alter this physical GRO-TDC resolution. Instead, the 7-bit LDO control word *ISW*⟨6:0⟩ adjusts the VTC charging current and hence *K_VTC_*, compensating the resulting voltage-to-code gain error. The nominal gain corresponding to the *t_LSB_* ≈ 3.9 ps reference condition is adopted as the target operating point.

Figure 13b expresses the 7-bit *ISW* tuning range in terms of the equivalent *t_LSB_* -drift range that can be compensated through VTC-gain adjustment.

The 3.08–4.77 ps range in Figure 13a was used as the worst-case statistical/PVT envelope for sizing the required gain-compensation range. The 7-bit *ISW* actuator in Figure 13b was then designed such that its VTC-gain tuning range covers the overall ADC gain variation corresponding to this entire *t_LSB_* envelope, with additional guard margin. Therefore, process and mismatch variations mainly change the final *ISW* code at which the overall voltage-to-code gain is restored, rather than eliminating the existence of a valid compensation point. This actuator-range margin provides robustness for the Stage-1 gain calibration.

To continuously stabilize the overall voltage-to-code gain under PVT variation, a mixed-signal closed-loop calibration architecture is proposed, as shown in Figure 14.

Following a hierarchical coarse–fine–ratio-lock strategy, the calibration loops are enabled in a staggered manner so that the fast gain acquisition establishes a valid operating range before the slower downstream loops dominate the correction. Stage 1 stabilizes the overall ADC gain and comprises two sub-steps—a successive-approximation-register (SAR)-based fast coarse lock followed by an accumulator-based background fine-tracking loop, while Stage 2 performs the *Tdither*/*t_LSB_* ratio lock. Together with the nonlinearity calibration of Section 3.4.2 (Stage 3), they constitute the three-stage background calibration of this work. Its central idea is to decouple in time the otherwise mutually coupled calibration objectives, allowing the fast upstream calibration to establish a valid operating range before the downstream loops become effective, thereby avoiding the mutual disturbance and convergence oscillation of conventional multi-error-coupled loops. The calibration compares the absolute output code *D_out_* against a configurable reference *REF*. For the 0.8-Vpp differential full-scale sinusoidal input used in the present evaluation, the expected magnitude of a signed 8-bit output gives:

(15) $$REF=E\left[\left|D_{out}\right|\right]=\left(2/\pi \right)\times 127\approx 80.85\to 81LSB$$

Equation (15) defines *REF* for the nominal full-scale sinusoidal condition used in the present evaluation. More generally, *REF* is a configurable digital parameter associated with the expected input amplitude and signal statistics of the target application, rather than a universal signal-independent constant. Once the input range is specified, *REF* can be configured accordingly and the Stage-1 loop tracks subsequent slow PVT-induced variations of the overall conversion gain. Therefore, the present implementation should be interpreted as a reference-assisted background gain calibration under a specified input envelope; it does not rely on an on-chip amplitude detector to autonomously distinguish a change in signal amplitude from a change in ADC gain. If the application input range is intentionally changed, the corresponding *REF* can be reconfigured before normal background tracking continues.

For the Stage 1 coarse lock, a statistical front end block-accumulates |*D_out_*| over an observation window of length *Nwin* = 1024. By the central-limit theorem, this reduces the statistical variance by about three orders of magnitude, lowering the standard deviation below 1 LSB to prevent misjudgment, while occupying only 1.024 μs at 1 GS/s—far shorter than the millisecond-scale PVT drift. The Stage-1 fine-tracking loop and the Stage-2 dither-ratio loop operate on a cycle-by-cycle basis to avoid windowing latency.

At power-up or reset, calibration first enters the Stage 1 coarse-lock phase. The control word is initialized to the midpoint 64 and resolved bit by bit from the MSB using a SAR logic. Within each bit’s window (*N_win_* = 1024), the error sum is accumulated:

(16) $$Sum\_e=\sum_{n=1}^{Nwin}\left(\left|D_{out}[n]\right|-REF\right)$$

The hardware decision is strictly based on the sign of *Sum_e*. If *Sum_e* > 0, the output codes are statistically too large, indicating that the effective voltage-to-code gain is above its target value. Consequently, the tested bit is retained as 1 to increase *ISW* and reduce *K_VTC_*; otherwise, the bit is cleared to 0. By binary search, all 128 levels are traversed in only seven window periods (about 7.2 μs), substantially shortening the initial convergence time. Figure 15 illustrates the transient convergence of this Stage 1 coarse-lock phase at 1 GS/s, where the average offset visually indicates the polarity of *Sum_e*.

After the initial coarse acquisition, the Stage-1 accumulator-based fine-tracking loop continuously tracks slow variations of the overall conversion gain caused by *t_LSB_* drift. To minimize hardware overhead and avoid the latency of windowed accumulation, this fine-tracking stage utilizes a sign-based Bang-Bang controller. The loop employs a 20-bit accumulator (*Acc*), where the upper 7 bits serve as *ISW* and the lower 13 bits act as fractional filtering bits to provide steady-state noise immunity.

Upon transitioning from the SAR phase, *Acc* is initialized to {*ISW_SAR_*<6:0>,1′b1,12′b0}. In each conversion cycle, *Acc* is updated based solely on the sign of the instantaneous error:

(17) $$e\left[n\right]=\left|D_{out}\left[n\right]\right|-REF,$$

(18) $$Acc\left[n+1\right]=Acc\left[n\right]+sign\left(e\left[n\right]\right),$$

where *e*[*n*] is the cycle-by-cycle error, and sign(⋅) denotes the sign function, which outputs +1 when *e*[*n*] > 0 and −1 otherwise. Here, *Acc* represents the 20-bit accumulator. Instead of accumulating the exact error magnitude, this sign-based Bang-Bang logic simply increments or decrements *Acc* by 1 in each clock cycle based on the polarity of *e*[*n*]. If the output code magnitude is too large (|*D_out_*| > *REF*), *e*[*n*] becomes positive, causing *Acc* to increment. This accumulation eventually carries over to the upper 7 bits, increasing *ISW* and thereby reducing *K_VTC_* until the gain error is minimized. This sign-based iteration effectively forms a low-pass filter with large inertia, fully suppressing steady-state chattering while eliminating the need for complex multipliers.

Concurrently with the Stage-1 fine gain tracking, the calibration proceeds to lock the relative ratio of the injected dither (*Tdither*/*t_LSB_*). The actual dither amount is precisely extracted by differencing the dither-enabled and dither-free output codes only in the cycles where dither is injected (*d*[*k*] = 1):

(19) $$ed\left[k\right]=|D\_END\left[k\right]-D\_EN\left[k\right]|-D\_target,\;\mathrm{when}d[k]\;=\;1$$

where *k* is the index of dithered cycles, and *D_target* = 8 LSB is the nominal target code increment. To drive *ed* to zero, a dedicated 8-bit accumulator (*Cdither*) is updated using a sign-based logic:

(20) $$Cdither\left[k+1\right]=Cdither\left[k\right]+sign\left(ed\left[k\right]\right)$$

The upper 4 bits of the 8-bit accumulator *Cdither* are extracted as *dithern*⟨3:0⟩ (i.e., Cdither⟨7:4⟩), and its bitwise inverse forms *ditherp*⟨3:0⟩, which directly drives the analog delay control terminals of the VDL. A larger *Cdither* increases *dithern*, which in turn decreases *ditherp* and reduces the delay *Tdither*. This Bang-Bang regulation ensures that the dither ratio is firmly locked without requiring a power-hungry LMS multiplier.

With the overall conversion gain already stabilized by Stage 1, the Stage-2 loop provides a stable operating reference for the dither ratio. The 8-bit length of *Cdither* acts as a deep digital filter; in the steady state, any residual toggling of the Bang-Bang controller is confined to a minimal step size. This corresponds to a femtosecond-level analog delay adjustment that is far below the TDC quantization noise floor (<0.5 *t_LSB_*). Such sub-LSB chattering is intrinsically absorbed by the quantizer, thereby keeping the residual dither-ratio variation below the TDC quantization noise floor and providing a sufficiently stable probe for the subsequent nonlinearity calibration.

Following the coarse lock, Figure 16 depicts the steady-state behavior in Stage 2. The system smoothly transitions to the 20-bit sign-based fine tracking phase, while the 8-bit *Cdither* accumulator simultaneously locks the dither delay. Compared to traditional multi-error coupled loops, the proposed decoupled architecture combined with sign-based Bang-Bang control exhibits excellent convergence stability and minimal steady-state chattering.

It is worth highlighting that the calibration loops operate with different time constants. The Stage-1 SAR logic completes a full-scale coarse lock in only 7 × 1024 cycles (approximately 7.2 μs at 1 GS/s), while the accumulator-based the accumulator-based fine loop continuously tracks slower overall-gain variations caused by *t_LSB_* drift. The calibration time constants should therefore be interpreted as reacquisition times for persistent operating-point changes rather than as a mechanism for suppressing cycle-to-cycle supply transients. If a large and persistent deviation moves the overall conversion gain outside the fine-tracking range, the fast SAR search can be re-enabled without resetting the GRO-TDC or the main data path. Because the observer and data paths are decoupled, temporary errors in the downstream dither-ratio or nonlinear-calibration states do not corrupt the raw *D_EN* path. Once the target conversion gain is reacquired, the downstream calibration loops naturally re-adapt to the new operating point. In steady state, the residual Bang-Bang chattering is absorbed by the lower 13 fractional bits of the accumulator, keeping the physical 7-bit *ISW* control word stable.

#### 3.4.2. Nonlinearity Calibration Based on Dither-Modulated Threshold-Crossing Statistics and EWMA-LMS

Following the two-stage calibration in Section 3.4.1, the overall voltage-to-code gain has been restored to the target defined by the nominal *t_LSB_* ≈ 3.9 ps reference condition, and the dither-induced code increment *D_target* ≈ 8 LSB has been stabilized. Nevertheless, owing to the finite output impedance of the current-steering cells, the VTC still exhibits pronounced nonlinearity in the large-swing region. As established in Section 3.1.1, the fully differential structure cancels only the first-order even-mode current error; the residual nonlinearity comprises an odd-order (HD3) slope compression together with a residual higher-order even-mode (HD2) asymmetry arising from device and layout mismatch. Both components must be extracted and compensated in the digital domain. It should be noted that the Stage-1 VTC-gain adjustment also changes the physical VTC operating point and can therefore modify its residual nonlinear coefficients; this interaction is handled by the hierarchical timing of the calibration loops rather than assumed to be absent.

Rather than directly fitting the integral-nonlinearity profile in the code domain—which demands high-precision residues and is sensitive to small-signal noise—this work extracts the nonlinearity from the statistics of threshold-crossing events modulated by the single-bit dither. The algorithm, whose digital structure is shown in Figure 17, comprises four cascaded blocks: a four-quadrant dynamic crossing detector, even/odd indicator formation with dither demodulation, an EWMA filter feeding an LMS weight-update engine, and a polynomial compensator. Consistent with the observer–data-path decoupled architecture of this work, all crossing events are evaluated on the dithered observer code *D_END* (which carries the probe and therefore conveys the nonlinearity information), whereas the extracted polynomial weights are fed forward to the clean data code *D_EN*, so that the main signal path is never disturbed. Performing nonlinearity correction directly in the digital background, rather than through analog over-design, follows the methodology established for VCO-based converters [19].

A four-quadrant dynamic crossing detector monitors, between two successive observer samples *D_END*[*n* − 1] and *D_END*[*n*], whether the code trajectory crosses the positive or negative threshold ±*D_th*, and in which direction; a one-sample delay *z*^−1^ provides the previous sample *D_END*[*n* − 1] for this comparison. Four mutually exclusive crossing events are defined:

(21) $$C\_UP+\left[n\right]=1,\mathrm{when}\;D\_END\left[n-1\right]<+D\_th\mathrm{and}\;D\_END\left[n\right]\geq +D\_th,$$

(22) $$C\_DN+\left[n\right]=1,\mathrm{when}\;D\_END\left[n-1\right]>+D\_th\mathrm{and}\;D\_END\left[n\right]\leq +D\_th,$$

(23) $$C\_UP-\left[n\right]=1,\mathrm{when}\;D\_END\left[n-1\right]<-D\_th\mathrm{and}\;D\_END\left[n\right]\geq -D\_th,$$

(24) $$C\_DN-\left[n\right]=1,\mathrm{when}\;D\_END\left[n-1\right]>-D\_th\mathrm{and}\;D\_END\left[n\right]\leq -D\_th,$$

each being zero otherwise. Because the dither randomly perturbs the falling-edge code by a calibrated amount in the *d*[*n*] = 1 cycles, the probability of crossing a given threshold is governed by the local slope (local gain) of the VTC transfer curve at that threshold: the flatter the local slope—that is, the stronger the compression—the fewer crossings occur per unit dither. The crossing statistics thus form a slope-sensitive probe of the nonlinearity.

To separate the even- and odd-order nonlinearities, which affect the transfer curve in physically distinct ways, two orthogonal indicators are constructed from the four events:

(25) $$I\_even\left[n\right]=\left(C\_UP+\left(n\right)+C\_DN+\left[n\right]\right)-\left(C\_UP-\left(n\right)+C\_DN-\left[n\right]\right),$$

(26) $$I\_odd\left[n\right]=\left(C\_UP+\left(n\right)+C\_DN+\left[n\right]\right)+\left(C\_UP-\left(n\right)+C\_DN-\left[n\right]\right),$$

where (A + B) denotes logical A OR B. Here *I_even* is the difference between the crossing activity at the positive and negative thresholds: under a perfectly odd-symmetric VTC the local slopes at +*D_th* and −*D_th* are identical, the two crossing probabilities are equal, and *I_even* has zero mean; any HD2 asymmetry unbalances the two sides and drives the mean of *I_even* away from zero, its sign and magnitude encoding the direction and severity of the even-order error. By contrast, *I_odd* is the sum of the crossing activity at both thresholds, representing the total crossing density; HD3 slope compression reduces the overall local slope near full scale and hence the total crossing density, so *I_odd* carries the odd-order information.

To distill the nonlinearity gradient from these indicators—each contaminated by the input signal’s own statistics and by quantization noise—the indicators are correlated with a bipolar version of the dither sequence, *s*[*n*] = 2*d*[*n*] − 1 ∈ {−1, +1}, derived from the same on-chip PRBS that gates the *Tdither* injection:

(27) $$e2\left[n\right]=I\_even\left[n\right]\cdot s\left[n\right],$$

(28) $$e3\left[n\right]=I\_odd\left[n\right]\cdot s\left[n\right]$$

This synchronous demodulation is the key to background operation: the dither-correlated component, which carries the nonlinearity, is folded to DC, whereas the input-correlated component, being uncorrelated with the pseudo-random *s*[*n*], is pushed to high frequency and subsequently removed by the EWMA filter. Because *s*[*n*] = +1 in the dithered cycles and −1 in the dither-free cycles, the demodulation is mathematically equivalent to differencing the crossing statistics between injected and non-injected cycles, thereby yielding a zero-mean error that requires no external reference.

The instantaneous errors *e*2[*n*] and *e*3[*n*] are then smoothed by a first-order EWMA filter, a single-pole IIR low-pass of the form

(29) $$E_{i}\left[n\right]=\beta E_{i}\left[n-1\right]+\left(1-\beta \right)e_{i}\left[n\right],\;i\in \left\{2,3\right\},$$

where *e_i_*[*n*] is the instantaneous demodulated error and *E_i_*[*n*] is its EWMA-smoothed value. The coefficient 1 − *β* is set to 2^−4^, corresponding to *β* = 15/16, to provide sufficient noise averaging while retaining the required tracking speed. The power-of-two coefficient also enables a shift-based hardware implementation without a general multiplier.

In a practical digital implementation, a hardcoded threshold would cause the calibration loop to stall if the input signal swing falls below the predefined level. To guarantee a sufficient density of threshold-crossing events across various input levels while strictly minimizing the digital hardware overhead, this design employs an amplitude-adaptive threshold scheduling scheme. Specifically, a lightweight envelope detector monitors the peak absolute code |*D_out_*|*_peak_* over a given window. This detector is used only for Stage-3 threshold scheduling and is independent of the configurable *REF* employed by the Stage-1 gain calibration. The threshold *D_th* is then dynamically assigned using a three-tier hardware-friendly logic:

(30) $$D\_th=\left\{\begin{array}{l} 48,\;\left|D_{out}\right|peak\geq 96\\ 24,\;48\leq \left|D_{out}\right|peak<96\\ 12,\;\left|D_{out}\right|peak<48 \end{array}\right.$$

For the nominal 0.8-Vpp differential full-scale input (corresponding to an 8-bit output range of approximately ±128), *D_th* is set to 48 LSB, placing the threshold in a high-probability region of the large-swing sinusoidal input. As the signal amplitude decreases, *D_th* is discretely scaled down. Because the sub-thresholds (24 and 12) are exact power-of-two fractions of the base value 48, this multi-zone scheduling can be implemented seamlessly via simple logic right-shifts (i.e., dividing by 2 or 4) and a multiplexer. This approach inherently bypasses the need for power-hungry digital multipliers while avoiding calibration deadlock over the designed input-amplitude zones.

Accordingly, within the designed input-amplitude zones, input-amplitude variation primarily changes the density of threshold-crossing events rather than disabling the nonlinear-calibration mechanism; *D_th* is reduced with the detected signal envelope to maintain sufficient crossing activity.

The smoothed gradients *E*2 and *E*3 drive two independent fixed-step LMS accumulators that update the even- and odd-order weights:

(31) $$W2\left[k+1\right]=W2\left[k\right]+\mu 2\cdot E2,$$

(32) $$W3\left[k+1\right]=W3\left[k\right]+\mu 3\cdot E3$$

The step sizes *μ*2 and *μ*3 are both chosen as 2^−8^ (approximately 0.004). This value is optimized to ensure a fast convergence time of less than 100 μs while keeping the steady-state fluctuation sufficiently small. Furthermore, selecting a power-of-two step size allows the multiplications in the LMS loop to be implemented as simple 8-bit right-shift operations, thereby significantly saving digital hardware overhead. The iteration frequency for updating *W*2 and *W*3 is set to 10 MHz. The two loops are orthogonal—*I_even*, being a difference, is blind to the symmetric HD3, while *I_odd*, being a sum, averages out the antisymmetric HD2—so the even- and odd-order weights converge without cross-interference.

The converged weights are finally fed forward to the clean data path to reconstruct the inverse of the VTC transfer nonlinearity. Since the slope compression reduces the quantized code, the compensator adds back the second- and third-order terms:

(33) $$DLSB=D\_EN+W2\cdot {\left(D\_EN\right)}^{2}+W3\cdot {\left(D\_EN\right)}^{3},$$

where the odd term (*D_EN*)^3^ preserves its sign across the full scale and thus restores the symmetric HD3 compression at both extremes, while the even term, through the sign of *W*2, corrects the residual HD2 asymmetry. The compensated 7-bit magnitude code *DLSB*[6:0] is concatenated with the dither-independent sign bit *DMSB* (Section 3.2) to form the final linearized output *D_out_*.

The compensator requires only two small-wordlength multipliers and two adders, avoiding any high-order division or look-up table, in keeping with the low-power objective of this work. The first two calibration stages are comparatively lightweight and are implemented mainly with SAR/sign-decision logic and accumulators, whereas the multiplier-based arithmetic is concentrated in the Stage-3 EWMA-LMS and polynomial-compensation path. While LMS-based adaptive nonlinearity correction has recently been demonstrated for high-speed pipelined ADCs in the voltage domain [20], this work extends the principle to a time-domain VTC front-end by combining single-bit dithering with the observer–data-path decoupled structure, thereby extracting the slope compression from threshold-crossing statistics without disturbing the main signal.

To quantify the convergence behavior of the third-stage nonlinearity calibration under the nominal 0.8-Vpp differential full-scale sinusoidal condition, Figure 18 illustrates the transient trajectories of the polynomial weights (*W*2 and *W*3) alongside the corresponding SFDR improvement. Operating at an iteration frequency of 10 MHz, the LMS engine updates the weights while the SFDR is evaluated every 10 μs. As shown, both *W*2 and *W*3 settle to their optimal steady-state values within 100 μs, lifting the SFDR from an initial uncalibrated state to over 66 dBc.

Combined with the 7.2-μs coarse lock in Stage 1 and the rapid Bang-Bang tracking in Stage 2, the complete three-stage calibration sequence achieves a total convergence time of approximately 110 μs, demonstrating rapid initial acquisition at the 1-GS/s sampling rate.

The calibration loops are hierarchically enabled and operate with separated time constants. Although the Stage-1 VTC-gain adjustment can also modify the residual VTC nonlinearity, the SAR-based coarse gain acquisition settles in approximately 7.2 μs, which is much faster than the approximately 100-μs nonlinear LMS adaptation. Consequently, the Stage-3 weights are primarily adapted after the VTC operating point has entered its valid range. During subsequent background operation, transient variations in the dither-correlated crossing statistics are averaged by the EWMA filter, whereas a persistent change in the VTC nonlinear characteristic produces a residual error that is continuously followed by the LMS adaptation. Therefore, after a sustained PVT-induced gain reacquisition, *W*2 and *W*3 can re-adapt to the nonlinear coefficients of the new VTC operating point without requiring a global reset.

## 4. Simulation Results

The proposed 1-GS/s 8-bit time-domain ADC was designed and verified in a 28 nm CMOS process. All results reported in this section are obtained from post-layout simulations including the extracted parasitic resistances and capacitances (R + C + CC) of the complete layout. Transient noise, clock jitter, PVT variations, and Monte-Carlo mismatch are considered where applicable. These results are intended to provide circuit-level verification of the proposed architecture rather than to substitute for silicon measurement.

The layout of the proposed ADC is shown in Figure 19. The whole design occupies a compact silicon area of 80 µm × 30 µm, corresponding to an active area of only 0.0024 mm^2^, which underlines its suitability for highly integrated micro-systems. The four functional blocks are laid out along the signal flow: the VTC front-end, the DIPG, the GRO-TDC, and the digital logic occupy successive regions from left to right, yielding a regular and routing-friendly floor-plan with short critical-path interconnects. This synthesized digital region includes the SAR/accumulator-based gain-control logic, the dither-ratio controller, the EWMA-LMS engine, and the polynomial compensator, and therefore already accounts for the principal digital overhead of the proposed calibration. It occupies approximately 39% of the total active core area. Importantly, the observer path does not require a duplicated GRO, because *D_EN* and *D_END* are obtained as two snapshots of the same physical oscillator, as described in Section 3.3. Specifically, the digital background calibration logic was fully synthesized and routed using standard cells, occupying an area of 31 µm × 30 µm (approximately 0.00093 mm^2^) at the rightmost end of the core.

Figure 20 summarizes the post-layout simulated power breakdown. Under the nominal condition, the ADC consumes 2.98 mW, with the DIPG, digital calibration logic, GRO, clock generation, TDC, and VTC accounting for 33.3%, 27.6%, 14.7%, 10.3%, 7.3%, and 6.8%, respectively. At the worst-power FF/1.1 V/125 °C corner, the total power increases to 3.89 mW, while the corresponding fractions become 29.6%, 29.8%, 16.7%, 12.1%, 6.6%, and 5.2%. Across the evaluated PVT range, the digital-calibration contribution varies from 22.3% to 29.8% and remains below 30% of the total ADC power.

To verify the effectiveness of the proposed three-stage digital background calibration, the output spectra were evaluated using a 16,384-point FFT with a 0.8-Vpp differential full-scale sinusoidal input at both a low input frequency and a near-Nyquist input frequency, as shown in Figure 21. In each case, the PSD before and after calibration is overlaid for direct comparison.

For a low-frequency input at Fin = 17 MHz, the calibration suppresses the dominant harmonic spurs—chiefly the second- and third-order harmonics (HD2 and HD3)—into the noise floor. As a result, the spurious-free dynamic range (SFDR) is improved from 53.1 dBc to 68.1 dBc, a gain of 15.0 dB, while the signal-to-noise-and-distortion ratio (SNDR) rises from 47.5 dB to 49.1 dB, corresponding to an effective number of bits (ENOB) of 7.86 bit.

At a near-Nyquist input of Fin = 487 MHz, where the VTC nonlinearity is most pronounced, the calibration remains highly effective: the SFDR is enhanced from 50.3 dBc to 67.0 dBc, an improvement of 16.7 dB, and the SNDR is raised from 41.6 dB to 46.0 dB, equivalent to an ENOB of 7.35 bit. The pronounced suppression of HD2 and HD3 at this high input frequency confirms that the dither-modulated nonlinearity calibration correctly extracts and compensates the large-signal slope compression of the VTC.

Consistently, the total harmonic distortion improves from −52.5 to −75.7 dB at 17 MHz and from −47.8 to −70.7 dB at 487 MHz after calibration, further confirming the suppression of the deterministic harmonic distortion.

To characterize the robustness of the calibration across the entire signal band, the SNDR and SFDR were swept over input frequencies from near-DC to the Nyquist frequency, with the results plotted in Figure 22.

The reported dynamic metrics also include transistor-level noise and clock-jitter effects. Full-chain input-referred noise was evaluated across PVT conditions, including the VTC front end, time comparator, and GRO-TDC contributions. The worst-case total rms noise is approximately 1.12 mVrms at the FF/LV/HT condition, with the GRO and time comparator providing the dominant contributions. Under the adopted 0.8-Vpp differential full-scale input, this noise level remains below one-half of the input-referred LSB. Sampling-clock jitter was extracted directly from circuit-level transient simulations rather than introduced through a behavioral jitter model. The variation across PVT corners is relatively small, with the worst-case rms jitter being approximately 796 fs at the SS corner. At Fin = 487 MHz, this jitter corresponds to an SNR limit of approximately 52 dB, which remains above the achieved 46.0 dB SNDR.

While the proposed digital background calibration successfully extracts and suppresses the deterministic large-signal nonlinearity of the VTC, it is worth noting that further improving the ADC precision by simply increasing the TDC interpolation factor or reducing the calibration step size would yield diminishing returns. This is because, once the deterministic VTC nonlinearity is compensated, the ultimate performance bottleneck shifts from the front-end transfer curve to the inherent stochastic noises and physical mismatches of the time-domain quantizer. Specifically, the GRO-TDC is fundamentally bounded by its intrinsic phase noise (jitter) during oscillation, the PVT-sensitive device mismatch within the sub-gate interpolation cells, and the uncalibratable broadband thermal noise. Consequently, rather than targeting high-resolution applications, this VTC-GRO decoupled architecture is strategically tailored for low-power and high-speed operation. It deliberately trades ultra-high absolute precision for exceptional energy efficiency and a highly compact silicon footprint, exactly fulfilling the requirements of the targeted highly integrated micro-systems.

To comprehensively verify the robustness of the proposed ADC under PVT variations, post-layout simulations were conducted across various extreme environmental conditions. Figure 23 illustrates the continuous performance sweeps across power supply (−5% to +10%) and temperature (−40 °C to 125 °C) variations.

Furthermore, to explicitly account for process variations, the dynamic performance of the ADC was evaluated across five typical process corners (TT, FF, SS, FS, SF) combined with extreme voltage (0.95 V/1.1 V) and temperature (−40 °C/125 °C) conditions. The comprehensive PVT post-layout simulation results are summarized in Table 1. Although the performance experiences some expected degradation under the worst-case extreme conditions (e.g., the SS corner with low supply and high temperature, yielding a near-Nyquist SNDR of >39.8 dB and SFDR of >52.2 dBc), the overall dynamic metrics remain highly stable and well within an acceptable range. This demonstrates that the proposed decoupled architecture and calibration algorithm possess strong stability and environmental adaptability across the full PVT spectrum.

Without calibration, both metrics degrade appreciably toward Nyquist, reflecting the increasing VTC nonlinearity at large input swings and high slew rates: the uncalibrated SFDR falls from 53.1 dBc at low frequency to 50.3 dBc near Nyquist, and the uncalibrated SNDR decreases from 47.5 dB at low frequency to 41.6 dB as the input frequency approaches Nyquist. After the proposed calibration is enabled, the SFDR is lifted to and maintained within the 67–68 dBc range across the full band (from 68.1 dBc at low frequency to 67.0 dBc near Nyquist), and the SNDR is improved to the 46–49 dB range (from 49.1 dB to 46.0 dB), remaining essentially flat with only a gentle roll-off toward Nyquist. The consistent improvement of roughly 15–17 dB in SFDR over the entire band demonstrates that the proposed calibration is not merely effective at a single tone but provides broadband, frequency-robust linearity enhancement up to the Nyquist edge, which is essential for the wideband acquisition required by the target micro-systems.

The static linearity of the proposed ADC was evaluated using the histogram method under a low-frequency (near-DC) full-scale sinusoidal input, so as to characterize the static transfer characteristic independently of dynamic settling effects.

As shown in Figure 24, before calibration the INL exhibits a pronounced S-shaped bow, with the two code extremes bending in opposite directions and the INL reaching −0.79/+1.41 LSB, while the differential nonlinearity (DNL) spans −0.65/+0.66 LSB. This S-shaped profile is the direct fingerprint of the slope compression introduced by the finite output impedance of the VTC current sources in the large-swing region, consistent with the odd-order (HD3-dominated) nonlinearity analyzed in Section 3.4.2. After the proposed polynomial nonlinearity calibration is enabled, the two end regions are flattened: the INL is reduced to −0.15/+0.70 LSB and the DNL to −0.34/+0.35 LSB, corresponding to roughly a 2× improvement in INL. No missing codes are observed across the entire output range, confirming that the calibration correctly extracts and compensates the VTC large-signal nonlinearity.

Thus, Figure 24 represents the equivalent end-to-end transfer linearity after the Stage-1 VTC gain adjustment and the Stage-3 digital compensation. The pronounced reduction of the original S-shaped INL directly indicates that the residual large-signal slope compression of the VTC has been effectively compensated, although the Stage-3 correction itself does not physically alter the VTC transfer curve.

Table 2 summarizes the post-layout simulated performance of the proposed ADC and compares it with recently reported state-of-the-art time-domain, hybrid, and SAR-based converters operating at gigasample-per-second rates. It should be noted that while the reference works report silicon measurement results, the performance of this work is evaluated based on rigorous post-layout simulations. To improve simulation fidelity, comprehensive parasitic resistance and capacitance (R + C + CC) extraction was included, together with transient noise and PVT variations. Although silicon measurement may introduce additional board-level and package-related non-idealities, the post-layout verification presented here provides circuit-level evidence for the effectiveness and hardware overhead of the proposed decoupled calibration architecture. For a conservative comparison with the measured designs in Table 2, the worst-case simulated power of 3.89 mW at the FF/1.1 V/125 °C corner is used for this work rather than the nominal 2.98 mW value. Using the same Walden-FoM definition, this corresponds to a FoM of approximately 23.9 fJ/conversion-step. These post-layout simulated results indicate a competitive combination of linearity, energy efficiency, and active area; however, direct quantitative comparison with measured silicon results should be interpreted with caution because the present work has not yet been experimentally validated.

For this work, the power and FoM are conservatively calculated using the worst-power PVT value of 3.89 mW, whereas the listed SNDR and SFDR correspond to the nominal post-layout condition.

Although comprehensive post-layout simulations have been conducted, actual silicon measurements may introduce additional physical non-idealities. For instance, high-frequency clock jitter can degrade the signal-to-noise ratio, which necessitates a low-jitter off-chip clock source during future testing. Furthermore, power supply noise is intrinsically mitigated by the proposed fully differential architecture and on-chip decoupling capacitors, while systematic process gradients are addressed through common-centroid layout routing and dummy devices. Board-level noise, package parasitics, and measured clock/supply disturbances cannot be fully represented by the present on-chip post-layout simulations and therefore remain limitations of this study. Their impact should be evaluated in a future silicon prototype.

## 5. Conclusions

This paper has presented a 1-GS/s 8-bit time-domain ADC in 28 nm CMOS targeting highly integrated micro-systems, which has been comprehensively verified via post-layout simulations. To overcome the deeply coupled *t_LSB_* drift and VTC nonlinearity that hamper conventional high-speed time-domain converters, an observer–data-path decoupled architecture was introduced, in which a single-bit dither is confined to an observer path for error extraction while the main signal is reconstructed in a dither-free data path, so that calibration is achieved without sacrificing the signal SNR. On this basis, a constant time-offset injection logic removes the GRO-TDC quantization dead-zone, and a three-stage digital background calibration compensates the ADC gain error induced by TDC-resolution drift through VTC-gain tuning, regulates the non-integer dither ratio via Bang-Bang control, and suppresses the residual even- and odd-order nonlinearity by extracting second- and third-order polynomial coefficients from dither-modulated threshold-crossing statistics through an EWMA-LMS engine. The hierarchically enabled calibration loops operate with separated time constants, allowing the fast gain acquisition to establish a valid operating point while the downstream dither-ratio and nonlinear-calibration loops subsequently adapt in the background. This organization suppresses the cross-interference typical of coupled multi-parameter calibration. Post-layout simulation demonstrates a Nyquist SFDR of 67.0 dBc, an SNDR of 46.0 dB, and an INL of −0.15/+0.70 LSB, with a nominal power consumption of 2.98 mW and a worst-case PVT power of 3.89 mW. Silicon measurement remains necessary to quantify package-, board-, and measurement-interface-related non-idealities beyond the scope of the present post-layout study.

## Figures and Tables

**Figure 1:**
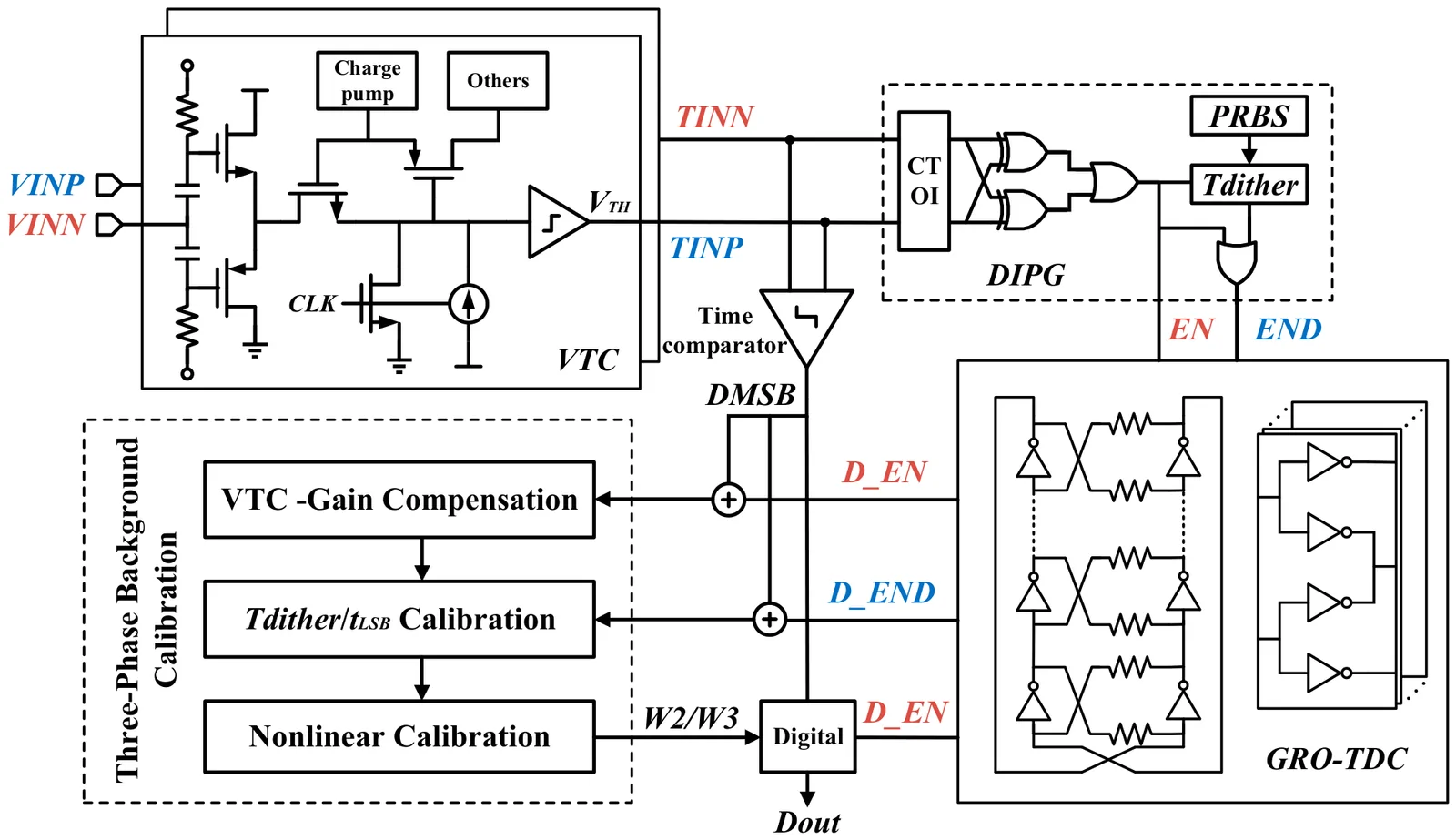
Overall architecture of the proposed ADC.

**Figure 2:**
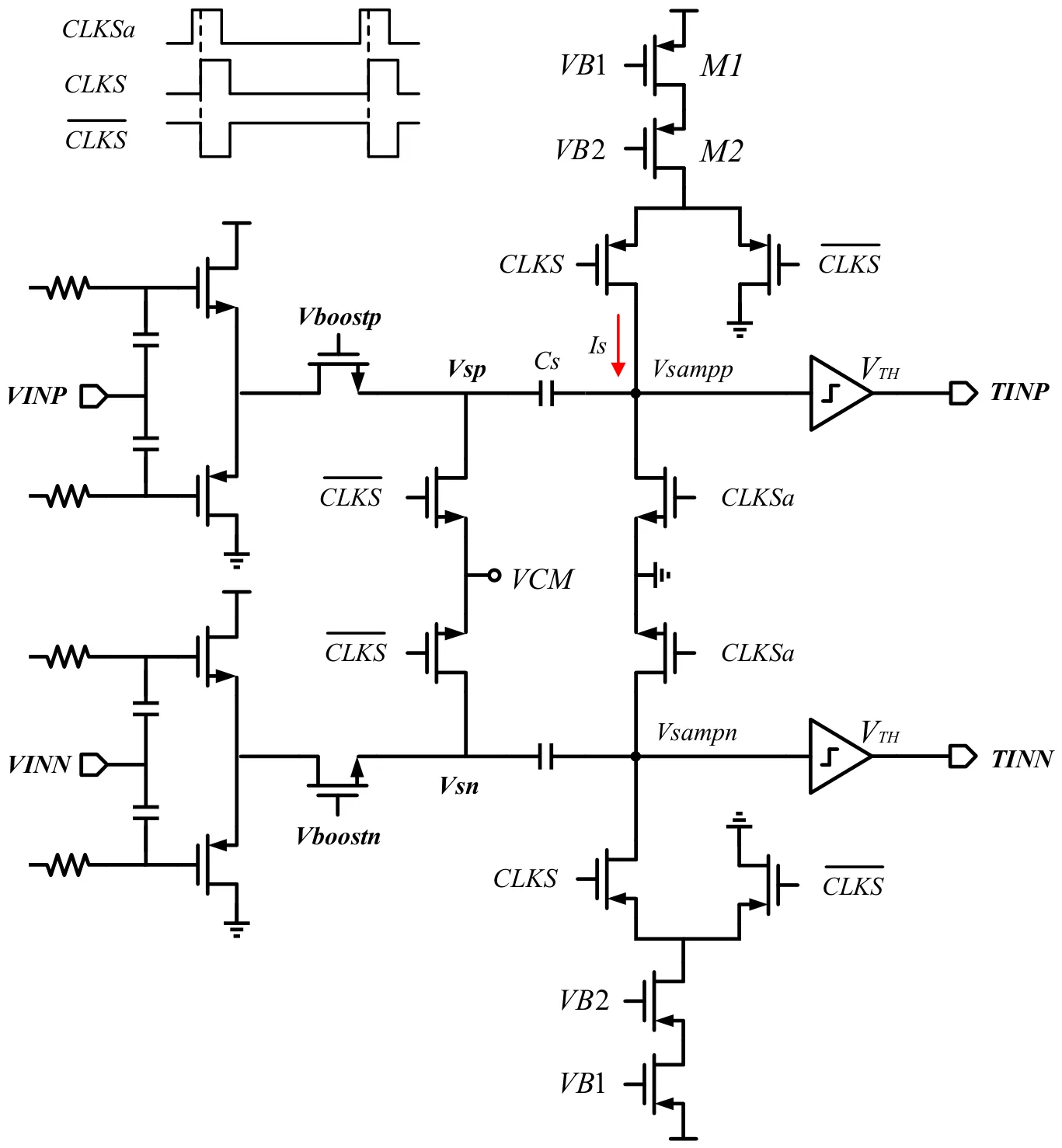
VTC front-end circuit.

**Figure 3:**
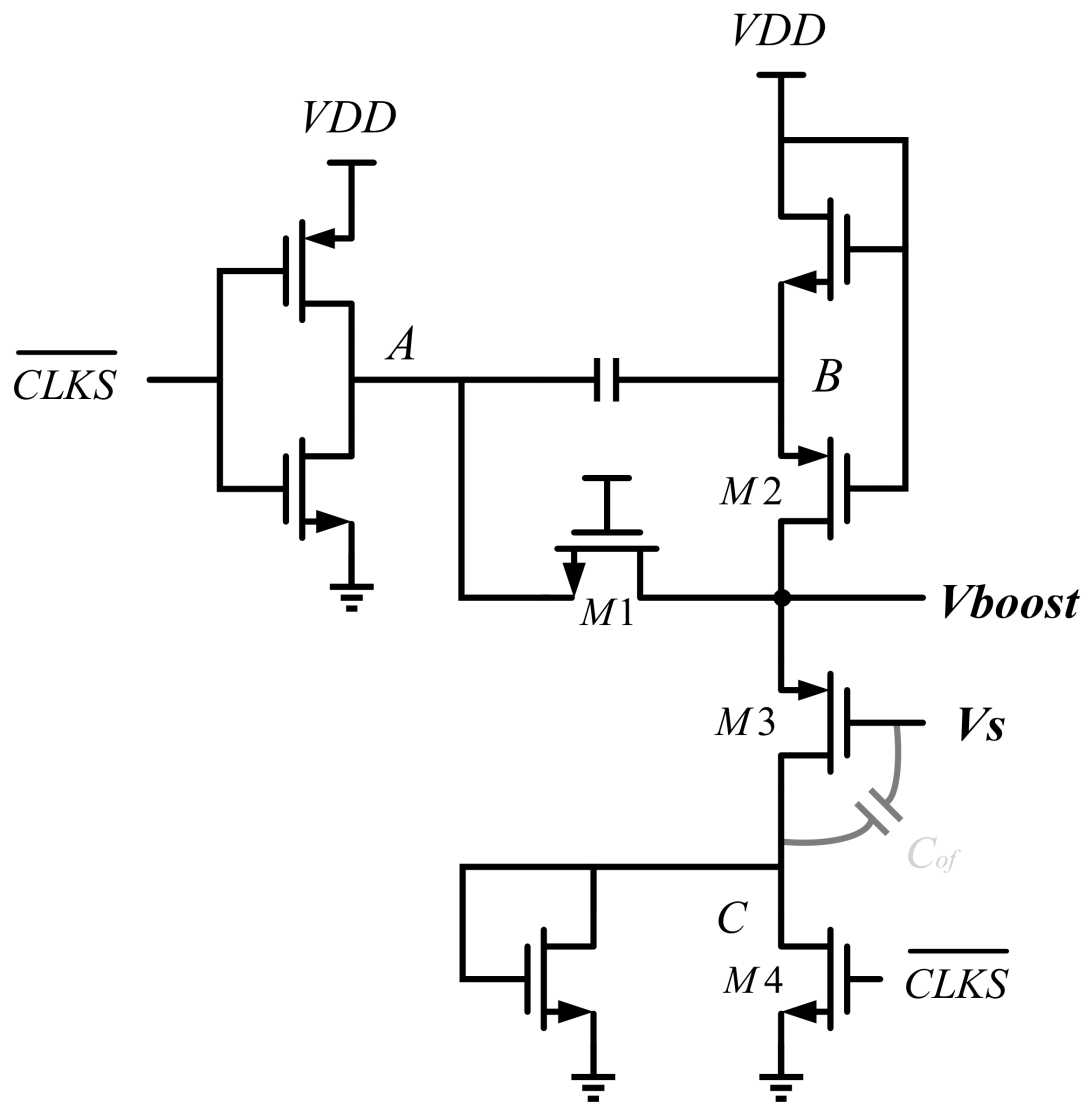
Gate-bootstrapping circuit.

**Figure 4:**
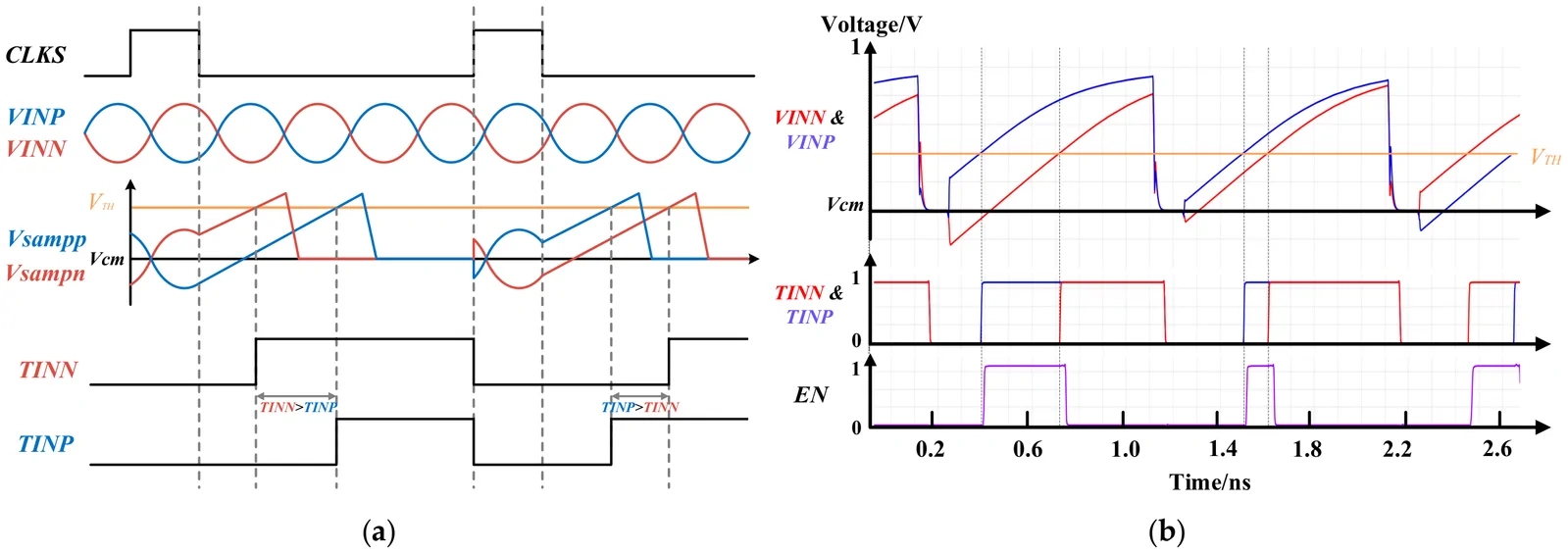
VTC conversion waveforms: (**a**) conceptual sampling and charging timing diagram; (**b**) post-layout simulation curves of the VTC front-end.

**Figure 5:**
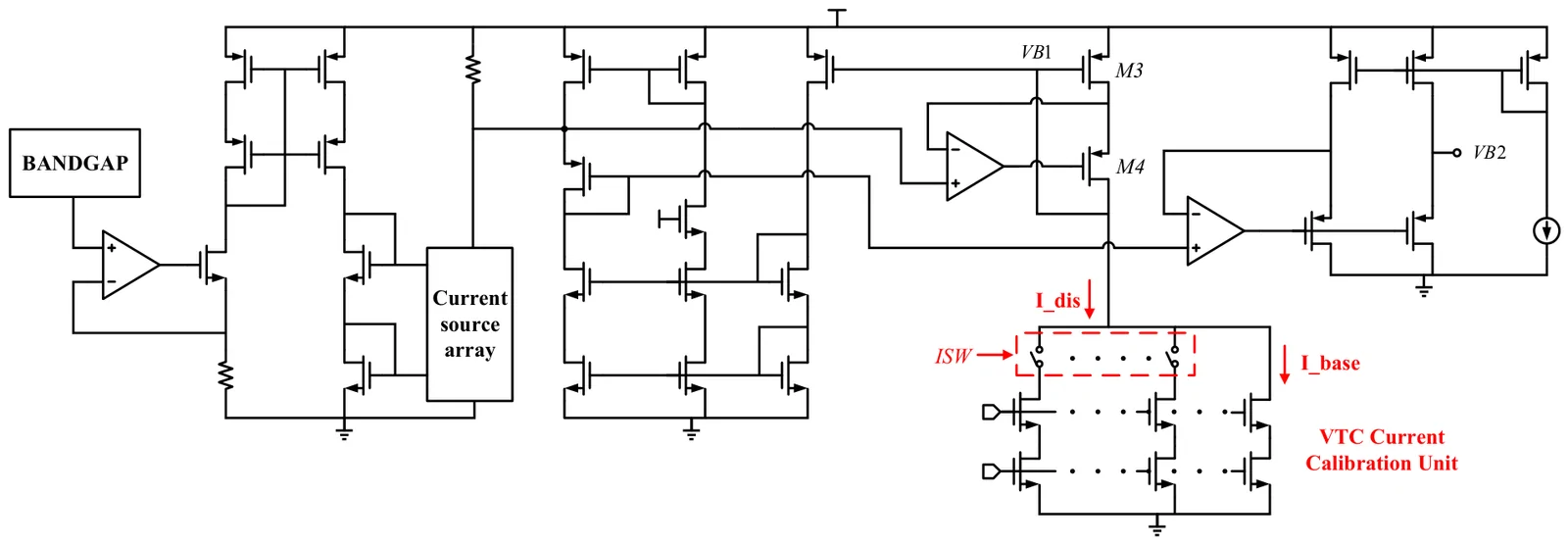
A 7-bit current-controllable LDO circuit.

**Figure 6:**
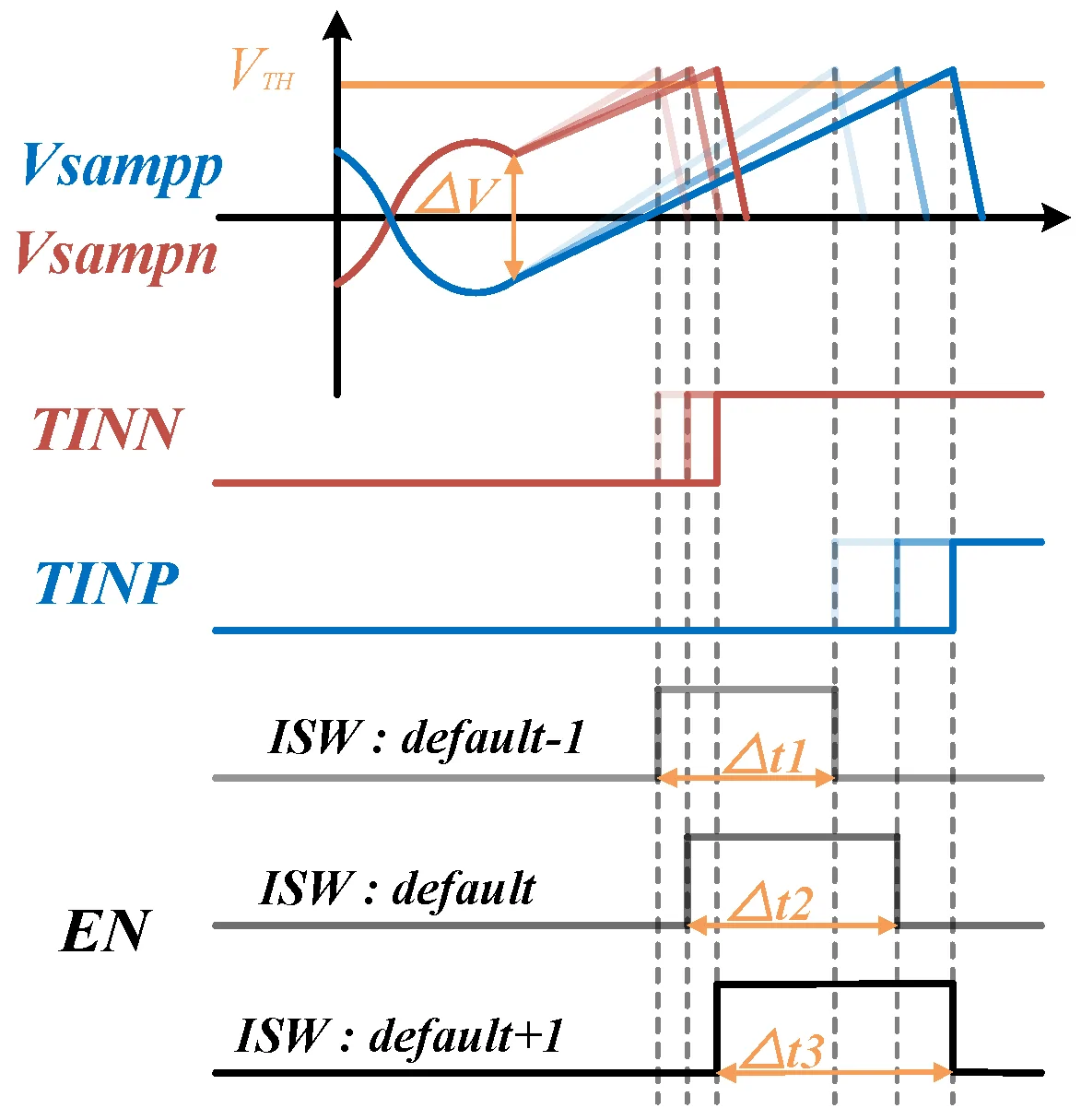
Conceptual relationship between the VTC conversion gain *K_VTC_* and the charging current *I_S_*, illustrating the inverse dependence used for overall ADC gain compensation.

**Figure 7:**
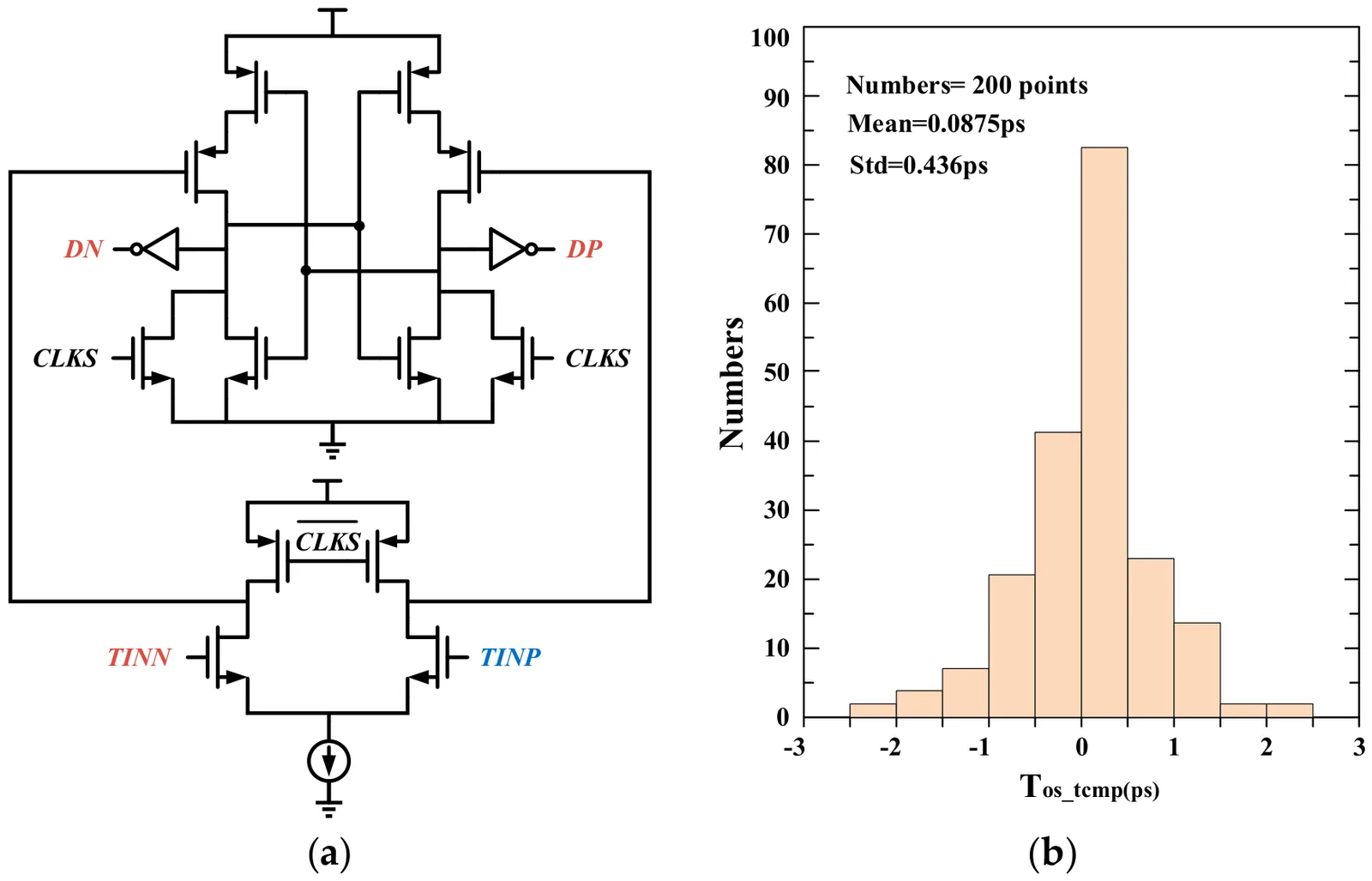
(**a**) Time comparator circuit; (**b**) 200-sample end-to-end Monte-Carlo equivalent-time-offset simulation including the current-controllable LDO-biased VTC and the time comparator.

**Figure 8:**
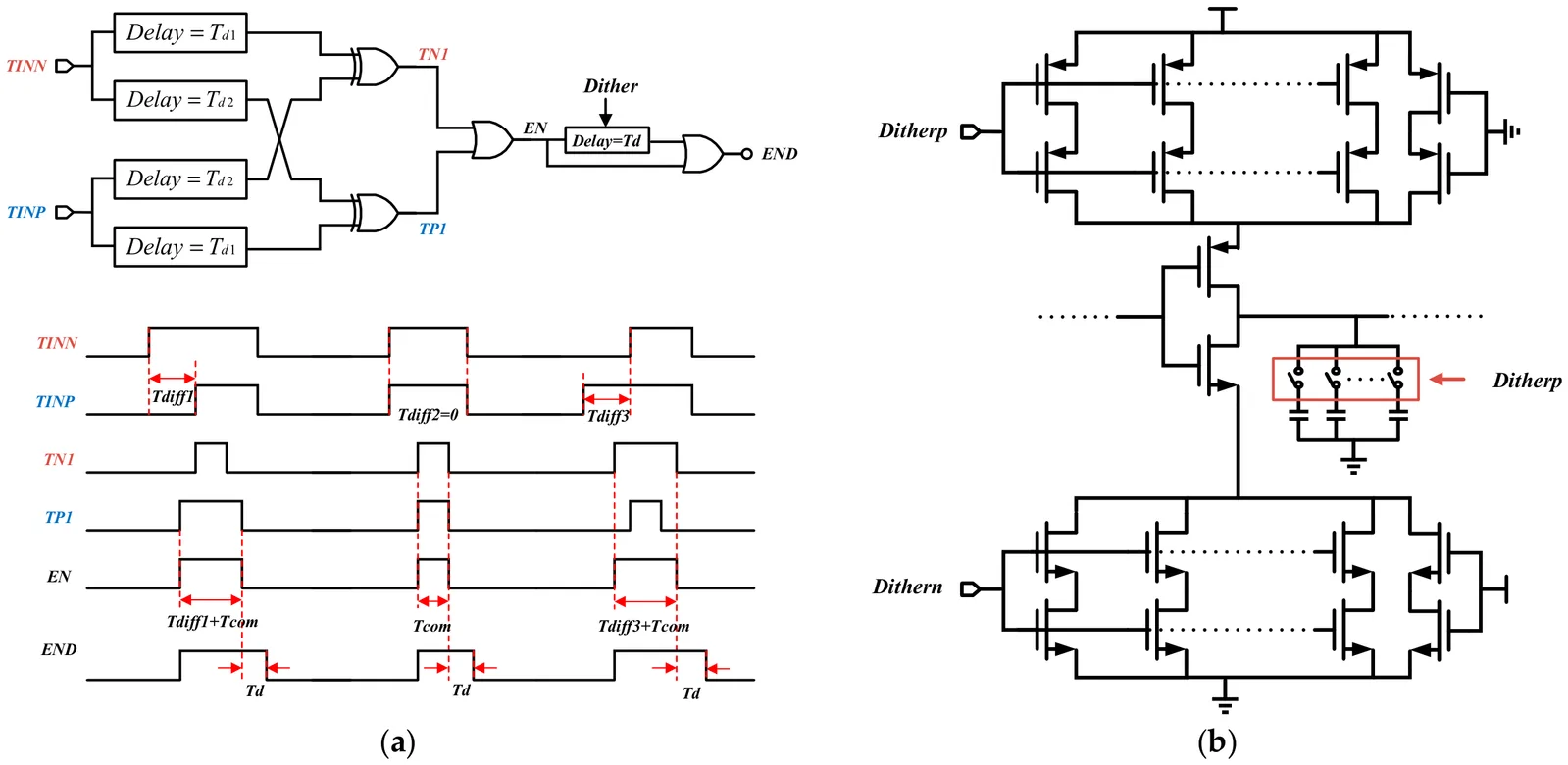
(**a**) DIPG pulse-generation logic and CTOI timing relation and the resulting EN/END waveforms; (**b**) delay-cell chain that generates the *Tdither* probe delay.

**Figure 9:**
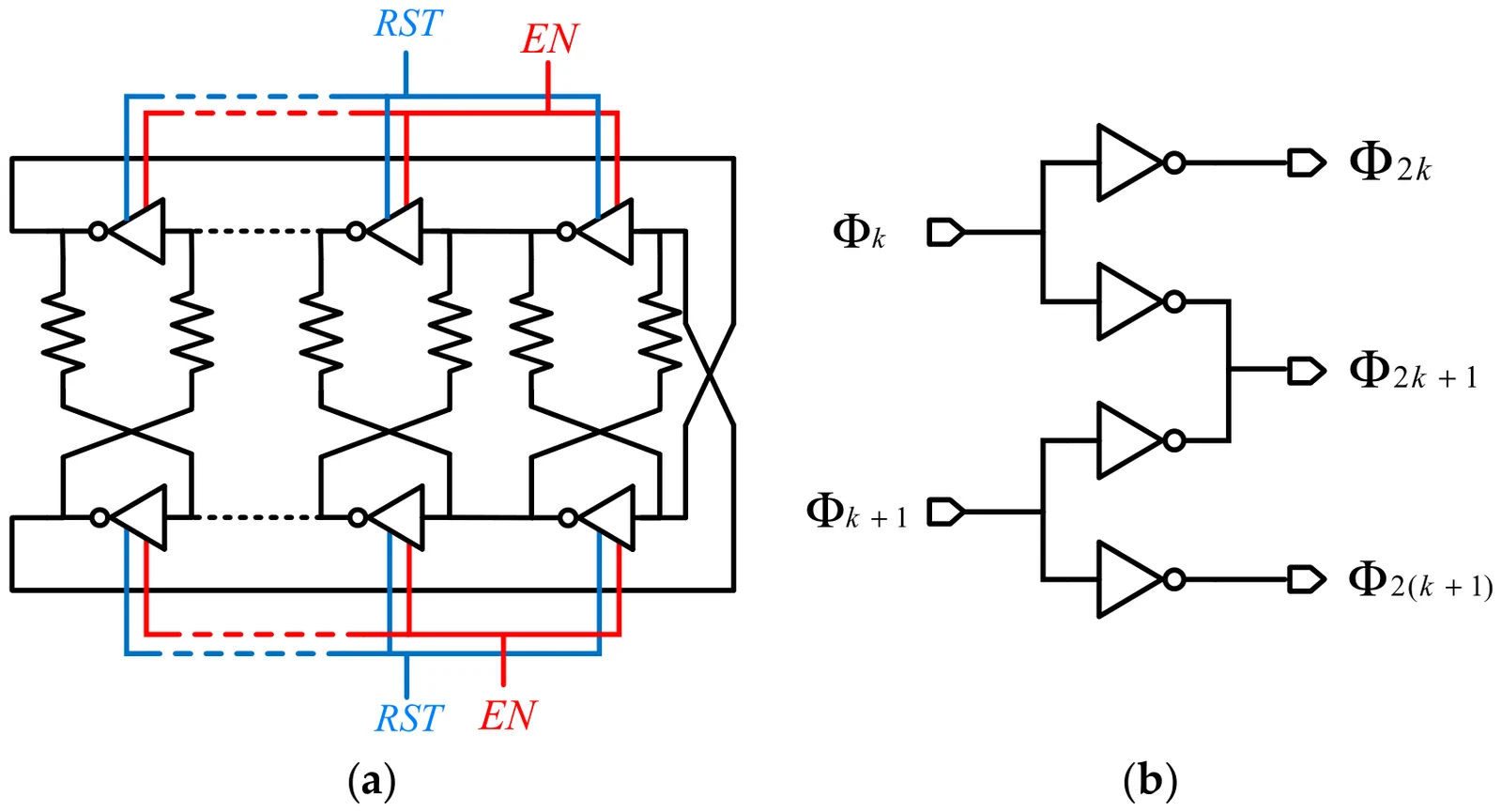
GRO-TDC circuit: (**a**) overall GRO circuit; (**b**) interpolation cell.

**Figure 10:**
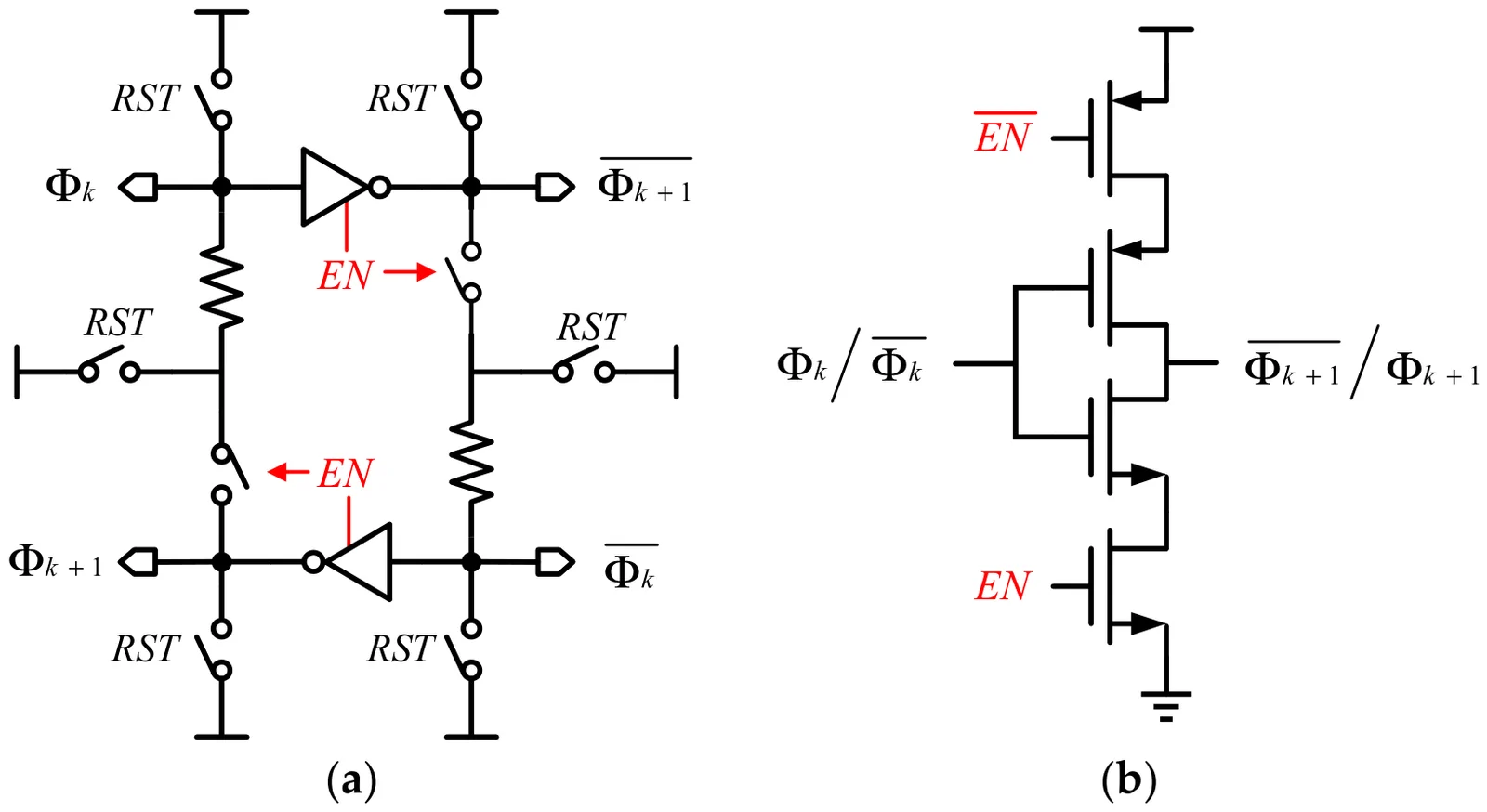
Basic cells of the GRO: (**a**) GRO oscillation cell; (**b**) resettable inverter.

**Figure 11:**
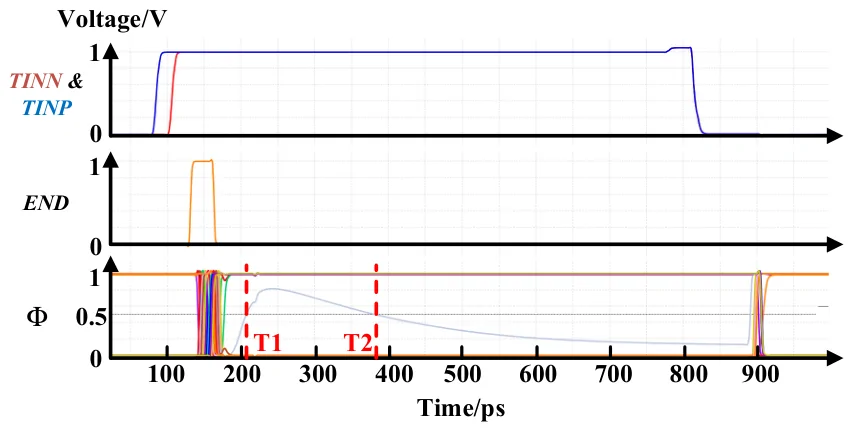
Illustration of the GRO stop instant and the sampling window (T1–T2).

**Figure 12:**
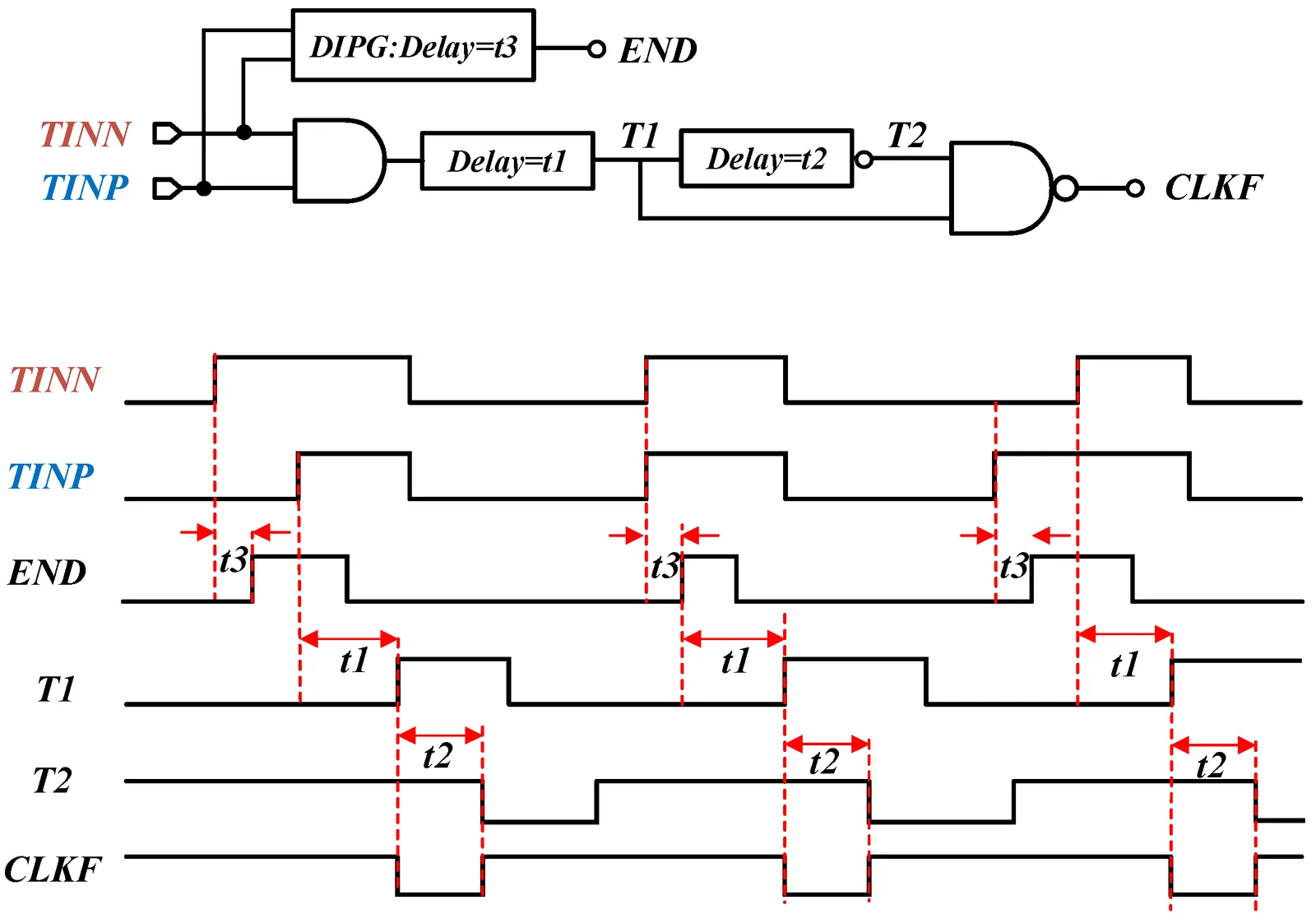
Phase-tracking sampling-clock generation circuit and its timing.

**Figure 13:**
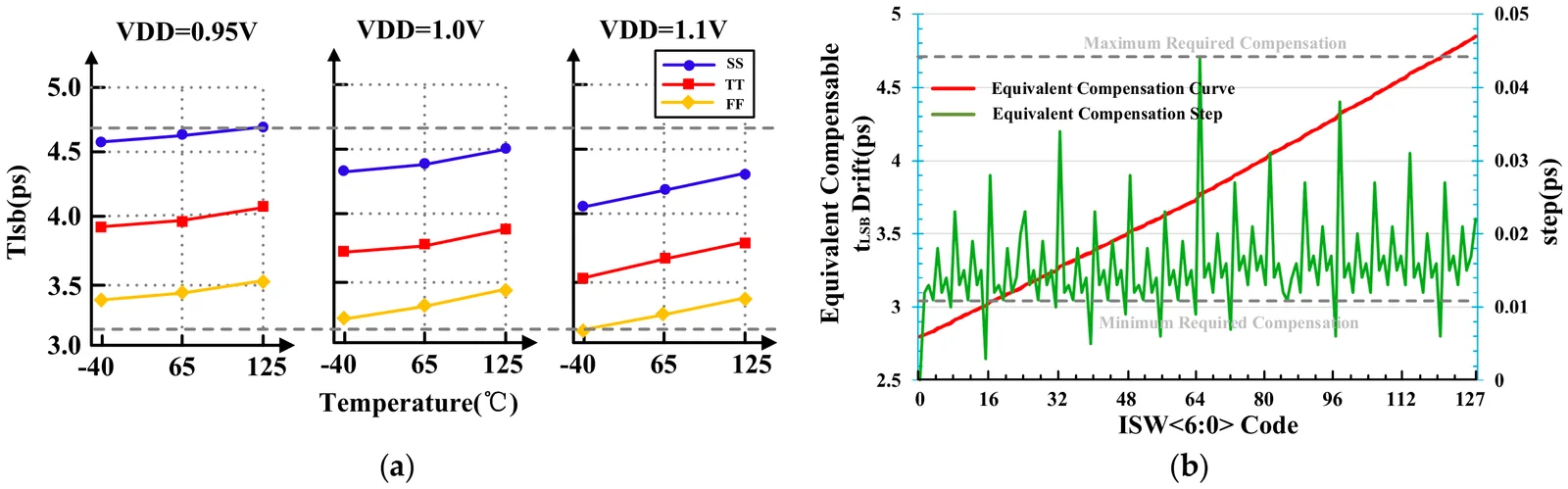
PVT-induced GRO-TDC resolution drift and the corresponding VTC-gain compensation range: (**a**) physical GRO-TDC *t_LSB_* across PVT corners (3.08–4.77 ps); (**b**) 7-bit *ISW* tuning range expressed as the equivalent *t_LSB_*-drift range that can be compensated through VTC-gain adjustment. The ordinate in (**b**) is an equivalent representation for direct comparison with (**a**); ISW does not physically tune the GRO-TDC *t_LSB_*.

**Figure 14:**
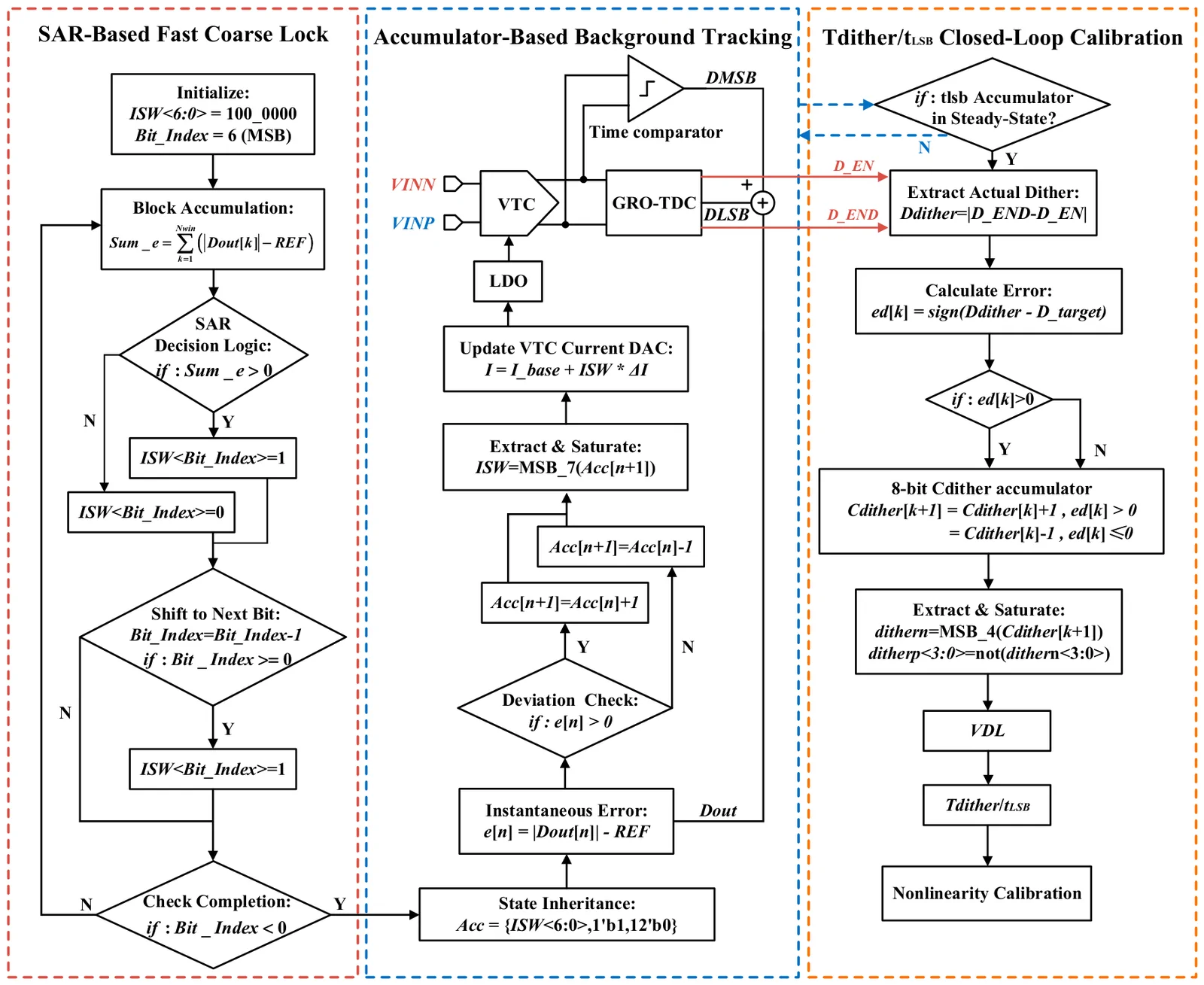
Mixed-signal closed-loop architecture for overall-gain calibration and *Tdither*/*t_LSB_* ratio lock.

**Figure 15:**
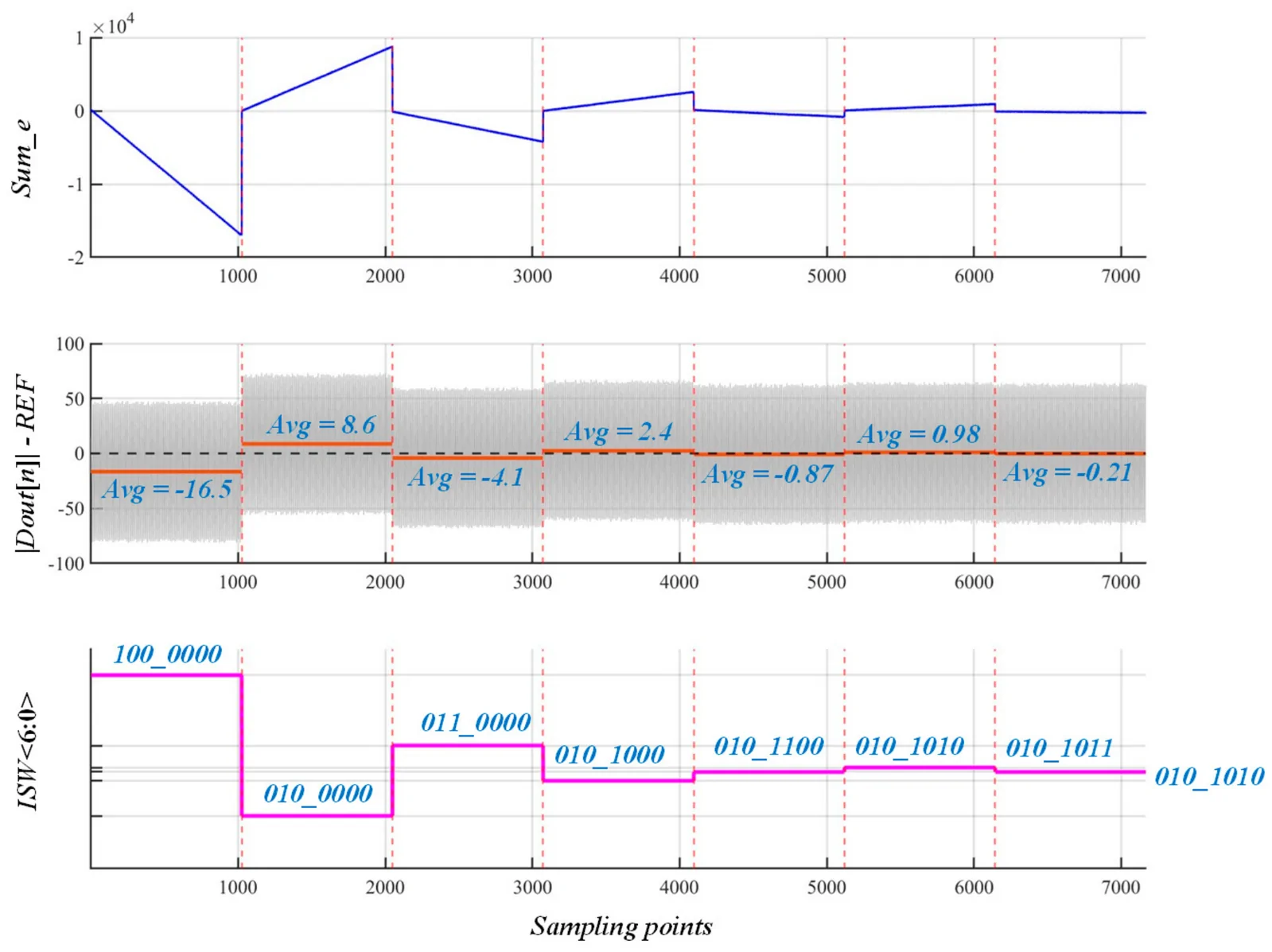
Transient convergence of the calibration loops at 1 GS/s (Stage 1): rapid coarse gain acquisition via windowed SAR logic.

**Figure 16:**
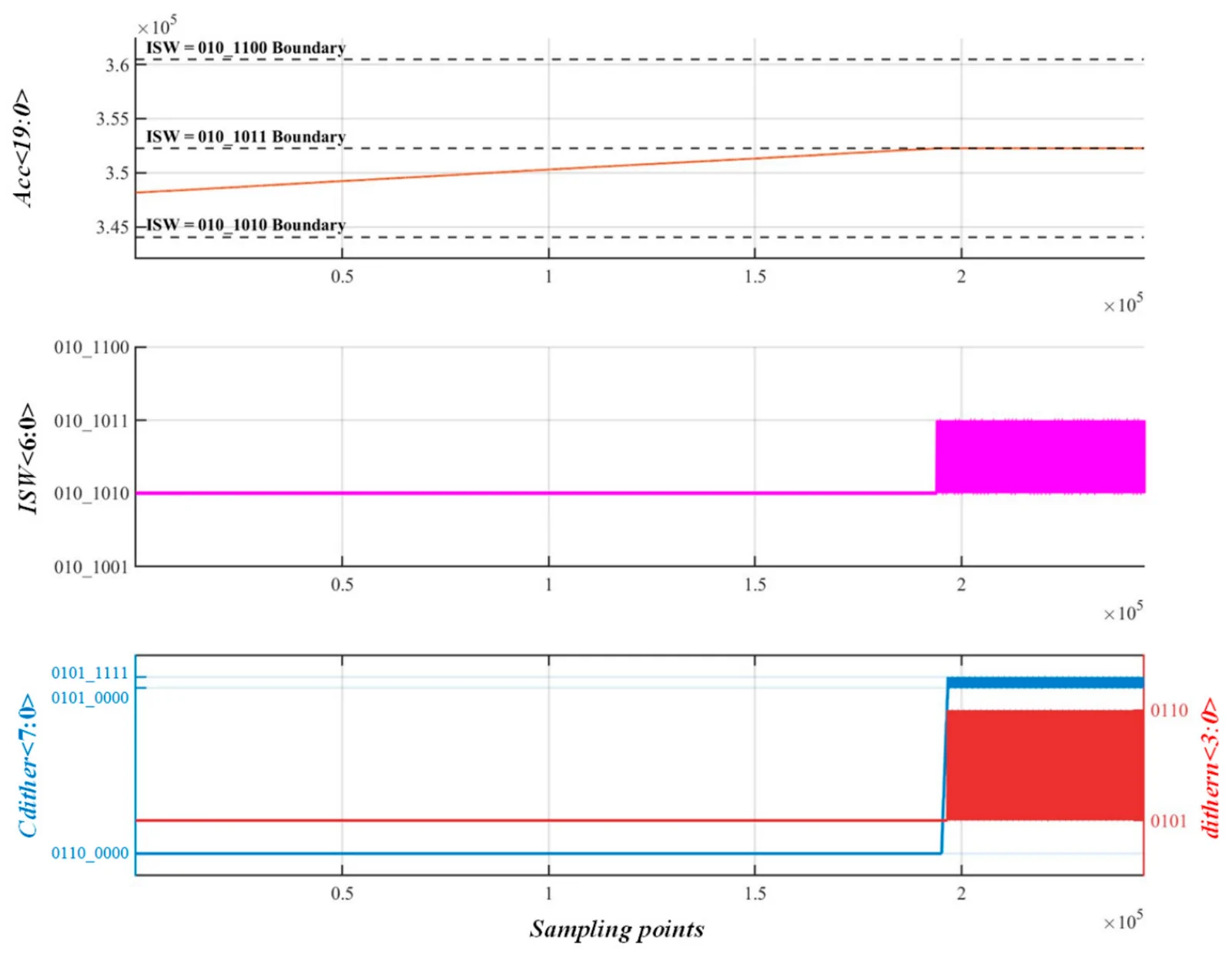
Transient convergence of the calibration loops at 1 GS/s (Stage 2): steady-state Stage-1 fine gain tracking and Stage-2 dither-ratio regulation via Bang-Bang accumulators.

**Figure 17:**
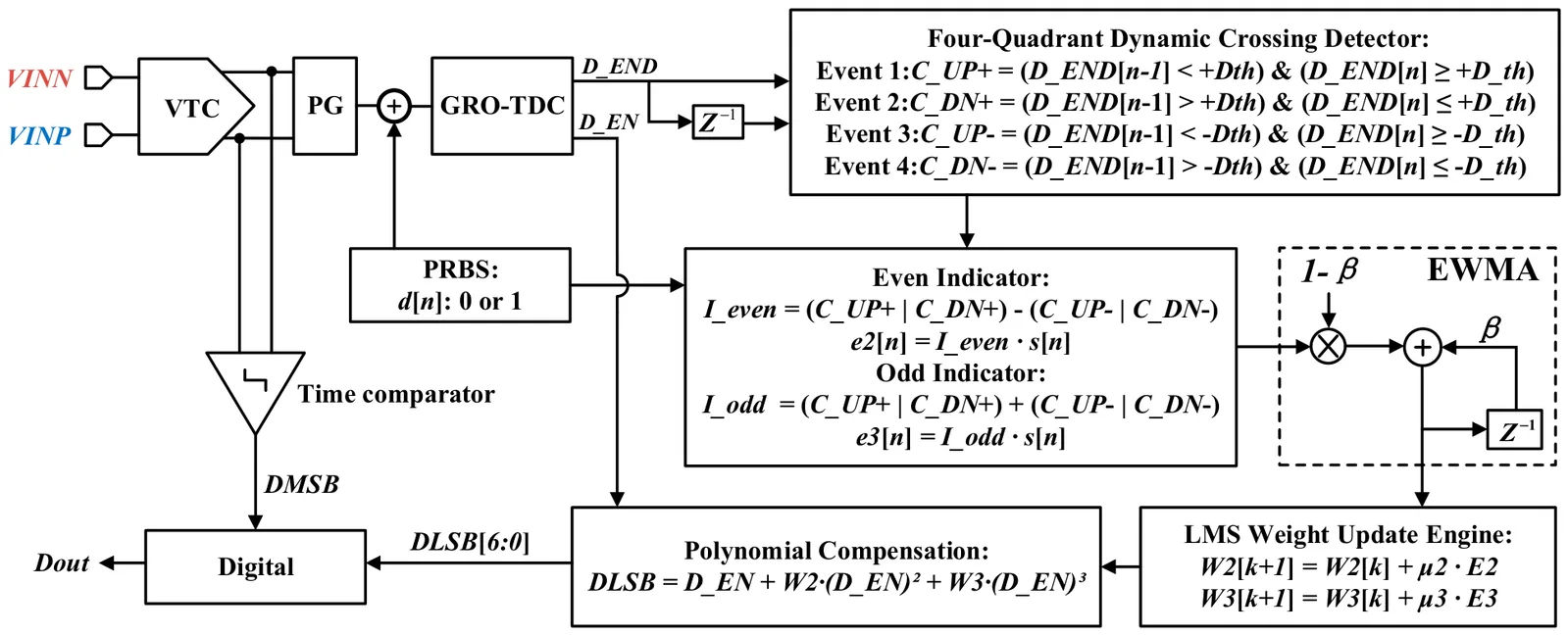
Block diagram of the nonlinearity calibration algorithm.

**Figure 18:**
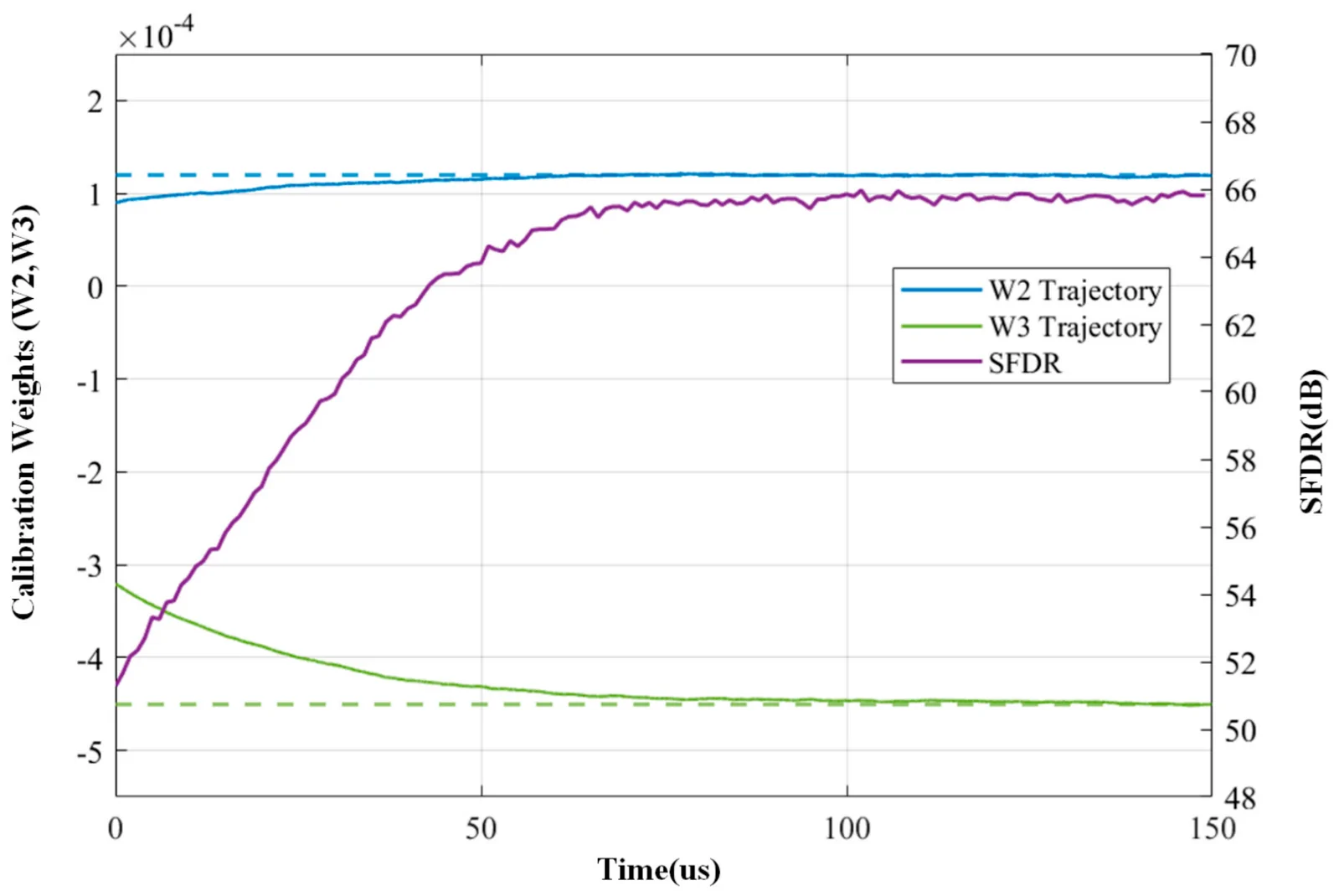
Transient convergence of the third-stage nonlinearity calibration, showing the trajectories of the polynomial weights (*W*2 and *W*3) and the corresponding SFDR improvement over time.

**Figure 19:**
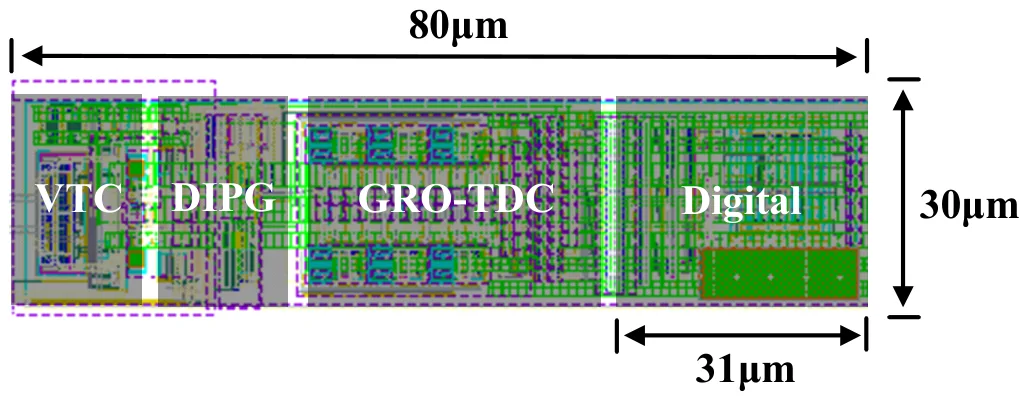
Layout of the proposed 1-GS/s 8-bit time-domain ADC.

**Figure 20:**
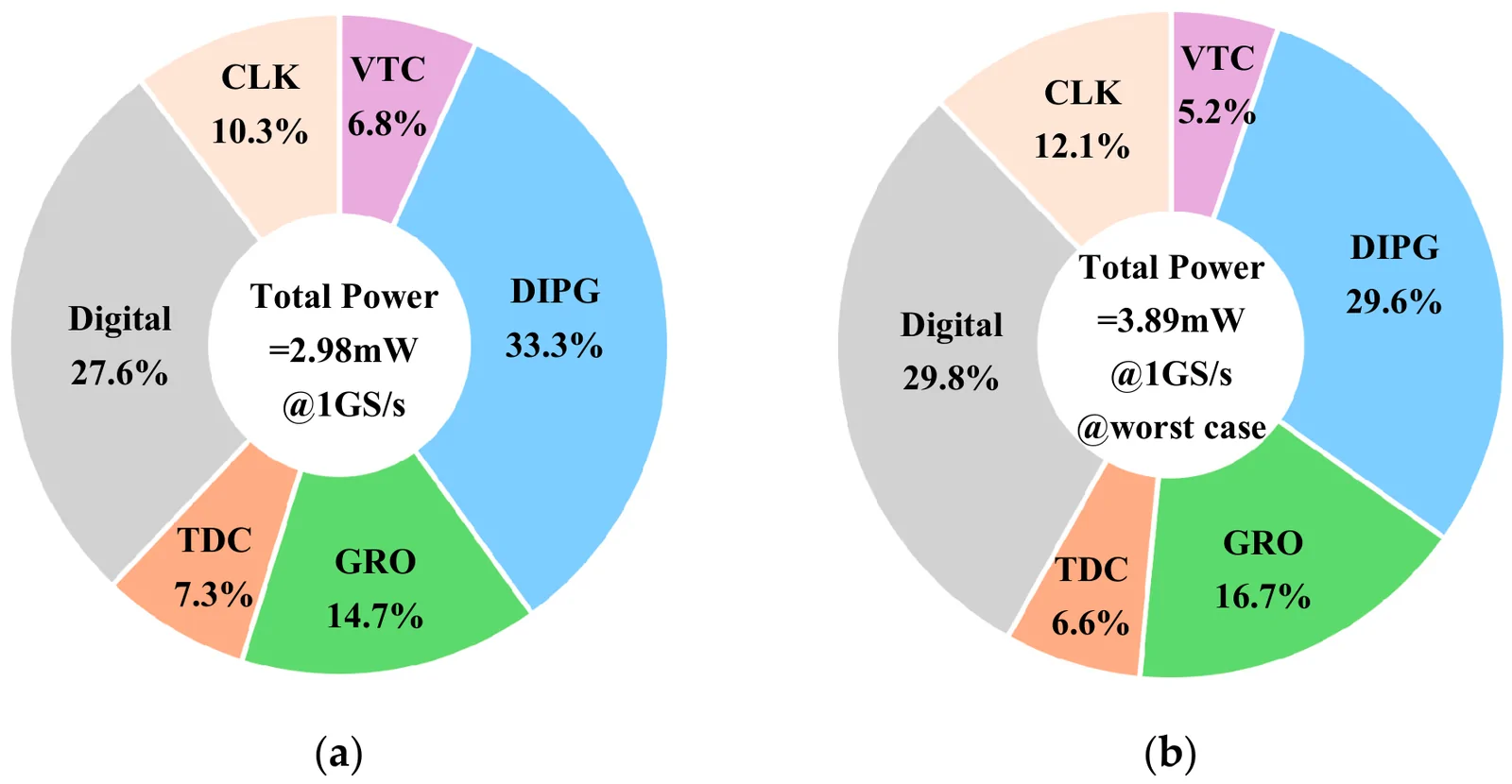
Post-layout simulated power breakdown of the proposed ADC at 1 GS/s: (**a**) nominal condition, total power = 2.98 mW; (**b**) worst-power FF/1.1 V/125 °C condition, total power = 3.89 mW.

**Figure 21:**
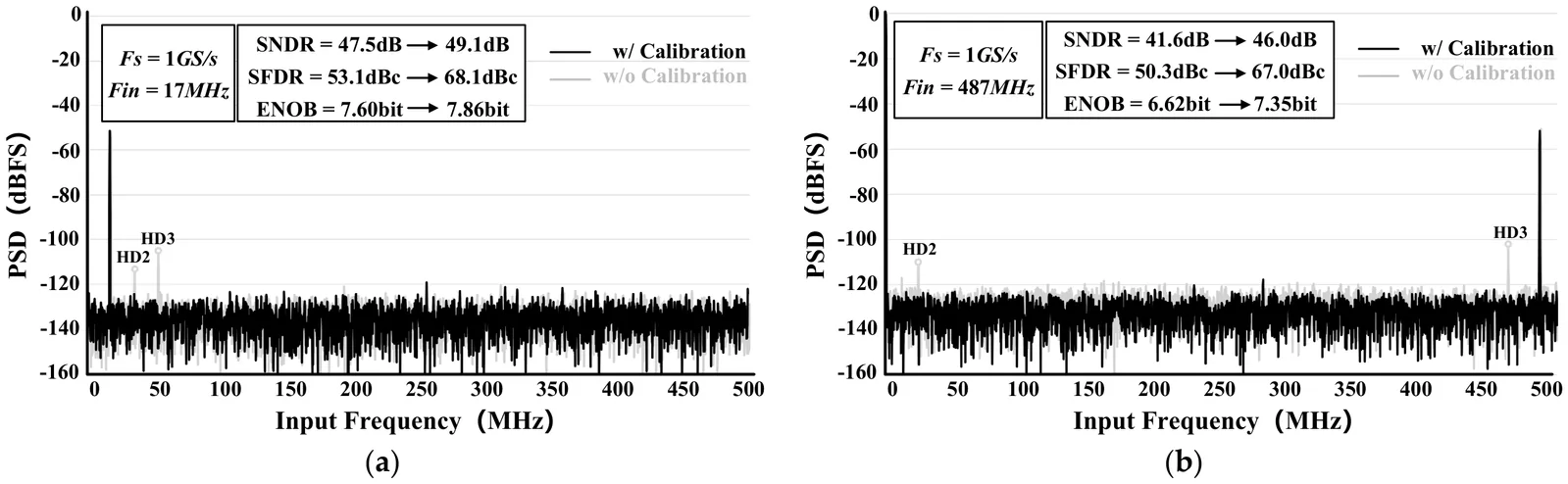
Output power spectral density (PSD) before and after calibration: (**a**) Fin = 17 MHz; (**b**) Fin = 487 MHz (near-Nyquist).

**Figure 22:**
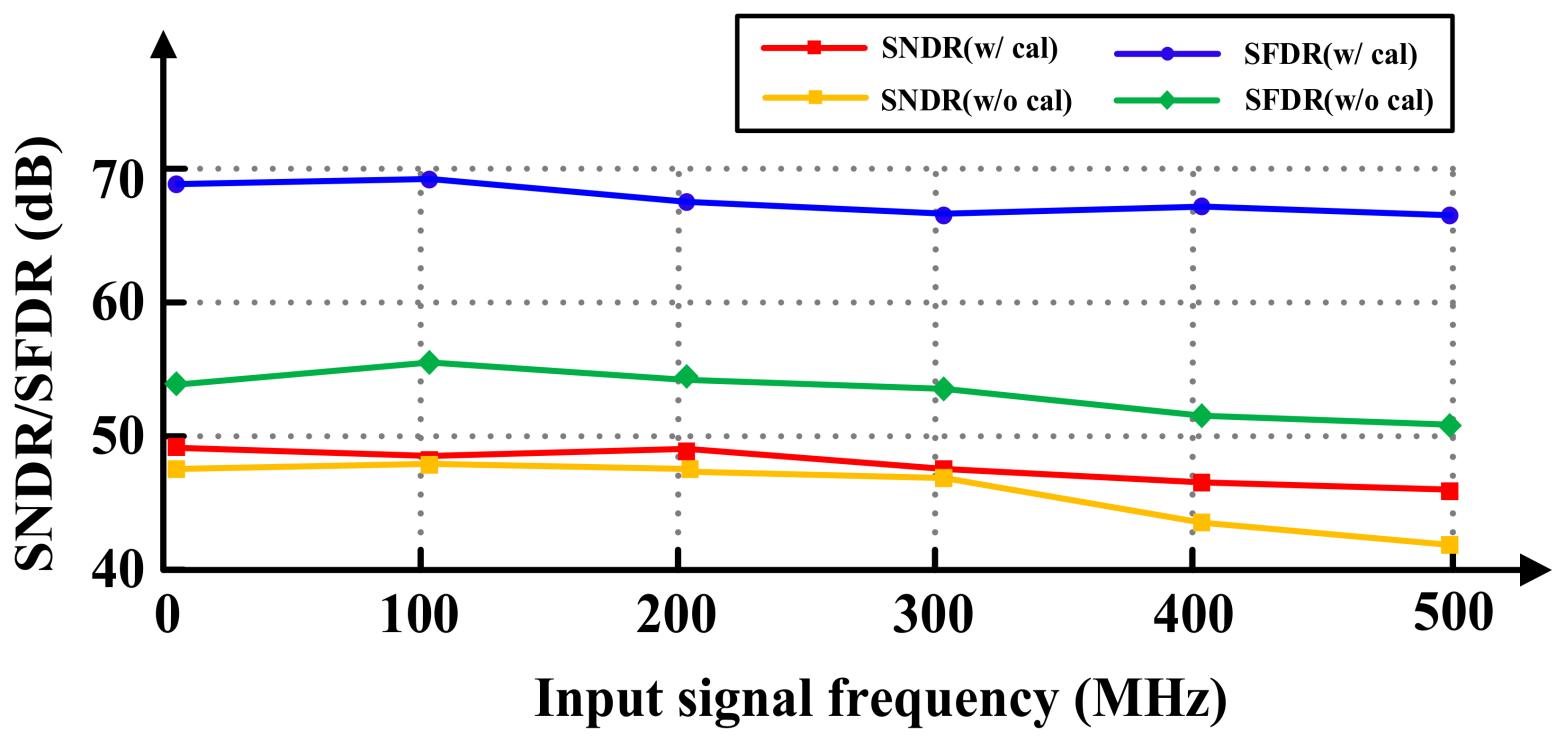
Simulated SNDR and SFDR versus input frequency, with and without calibration.

**Figure 23:**
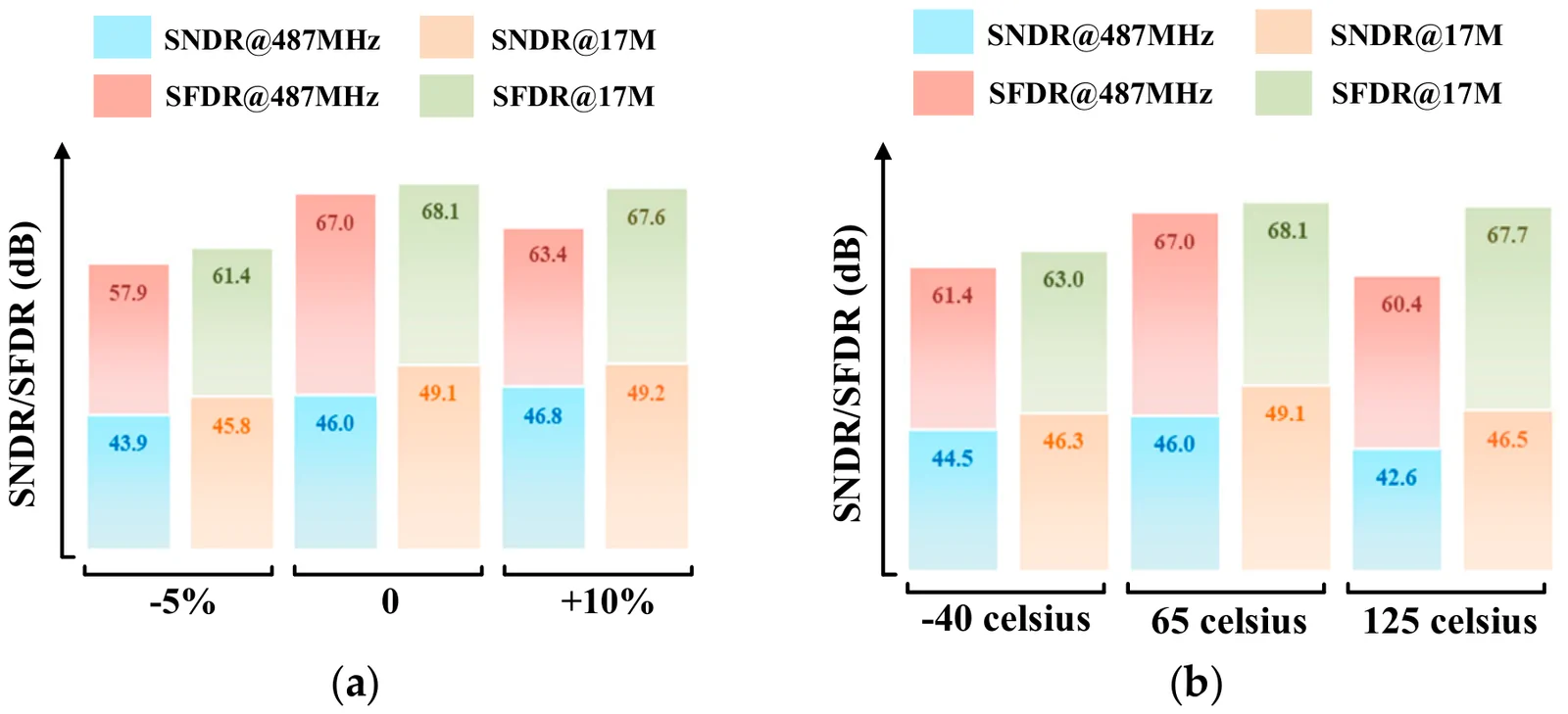
Post-layout simulated dynamic performance of the overall ADC under different PVT conditions. (**a**) Supply voltage variations (−5% to +10%). (**b**) Temperature variations (−40 °C to 125 °C).

**Figure 24:**
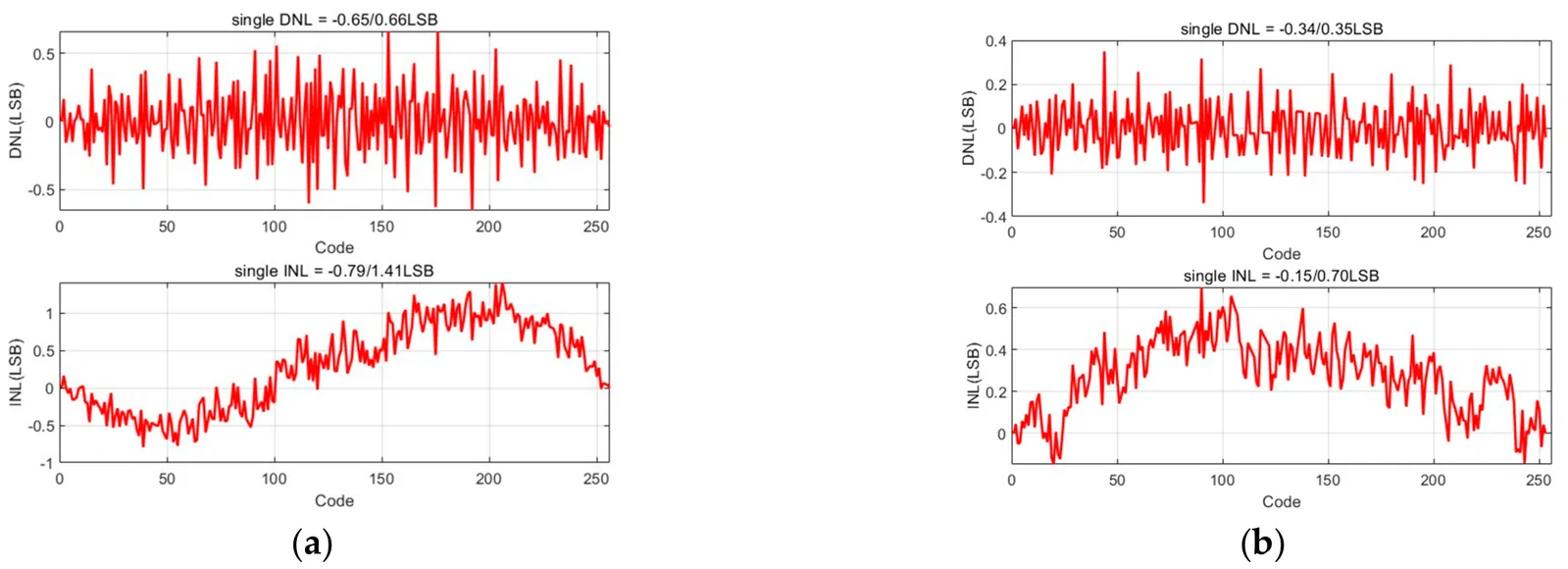
Static linearity of the proposed ADC: (**a**) without calibration; (**b**) with calibration.

**Table 1:** Post-layout simulated dynamic performance across PVT corners.

Reference	TT	FF	SS	FS	SF
Temperature (°C)	−40/125	−40/125	−40/125	−40/125	−40/125
Supply (V)	0.95/1.1	0.95/1.1	0.95/1.1	0.95/1.1	0.95/1.1
SNDR at Low-freq (dB)	>43.5	>42.2	>40.2	>42.8	>41.3
SFDR at Low-freq (dBc)	>61.1	>64.7	>54.9	>58.3	>61.7
SNDR at Nyquist (dB)	>42.3	>41.1	>39.8	>42.3	>40.4
SFDR at Nyquist (dBc)	>58.8	>61.6	>52.2	>54.4	>55.8

**Table 2:** Performance summary and comparison with state-of-the-art ADCs.

Reference	[14] *	[21]	[22]	[23]	[24]	[25]	This Work
Validation Method	Measured	Measured	Measured	Measured	Measured	Measured	Post Sim
Architecture	Two-Step	SAR-TDC	RNS-TDC	Vernier-TDC	SAR-Flash	GRO-TDC	GRO-TDC
Fs (GS/s)	1.0	1.0	2.0	0.9	1.0	2.0	1.0
Resolution (b)	8	8	8	8	8	7	8
Process	65 nm	12 nm	65 nm	65 nm	28 nm	28 nm	28 nm
Supply (V)	1.0	0.8	1.2/1.3	1.0	1.1	1.0	1
BW [Hz]	500M	10G	1.74G	450M	500M	1G	500M
SNDR at Nyquist (dB)	44.4	42.0	40.7	41.9	45.5	36.9	46.0
SFDR at Nyquist (dBc)	59.7	52.0	48.4	49.5	59.4	49.8	67.0
Calibration Type	No	Background	Offline (histogram-based)	No	Foreground	Foreground (on-chip)	Background (on-chip)
DNL/INL (LSB)	0.6/0.8	-	0.14/0.61	0.7/0.9	0.59/0.82	0.97/0.73	0.35/0.70
Power (mW)	2.3	2.6	21	3.5	2.55	3.65	3.89 (worst)
Area (mm^2^)	0.007	0.006	0.08	0.029	0.002	0.004	0.0024
FoM (fJ/conv.-step) **	18.7	2.53	100.9	37	16.6	31.4	23.9

* Sub-ADC results. ** FoM = Power/(BW × 2 × 2^ENOB^).

## Data Availability

The original contributions presented in this study are included in the article. Further inquiries can be directed to the corresponding author.

## References

[1] Murmann B. (2015). The Race for the Extra Decibel: A Brief Review of Current ADC Performance Trajectories. IEEE Solid-State Circuits Mag..

[2] Kim J., Jang T.-K., Yoon Y.-G., Cho S. (2010). Analysis and Design of Voltage-Controlled Oscillator Based Analog-to-Digital Converter. IEEE Trans. Circuits Syst. I Reg. Pap..

[3] Li H., Sin S.-W., Martins R.P., Guo M. Voltage-Domain vs. Time-Domain: Trade-offs in High-Speed Applications. Proceedings of the 2025 IEEE 16th International Conference on ASIC (ASICON).

[4] Li S., Mukherjee A., Sun N. (2017). A 174.3-dB FoM VCO-Based CT ΔΣ Modulator With a Fully-Digital Phase Extended Quantizer and Tri-Level Resistor DAC in 130-nm CMOS. IEEE J. Solid-State Circuits.

[5] Zhong Y., Li S., Tang X., Shen L., Zhao W., Wu S., Sun N. (2020). A Second-Order Purely VCO-Based CT ΔΣ ADC Using a Modified DPLL Structure in 40-nm CMOS. IEEE J. Solid-State Circuits.

[6] Kull L., Toifl T., Schmatz M., Francese P.A., Menolfi C., Braendli M., Kossel M., Morf T., Andersen T.M., Leblebici Y. (2013). A 3.1 mW 8b 1.2 GS/s Single-Channel Asynchronous SAR ADC With Alternate Comparators for Enhanced Speed in 32 nm Digital SOI CMOS. IEEE J. Solid-State Circuits.

[7] Chen Q., Liang Y., Kim B., Boon C.C. A 3GS/s Highly Linear Energy Efficient Constant-Slope Based Voltage-to-Time Converter. Proceedings of the 2020 IEEE International Symposium on Circuits and Systems (ISCAS).

[8] Xing X., Gielen G.G.E. (2015). A 42 fJ/Step-FoM Two-Step VCO-Based Delta-Sigma ADC in 40 nm CMOS. IEEE J. Solid-State Circuits.

[9] Tang X., Yang X., Zhao W., Hsu C.K., Liu J., Shen L., Mukherjee A., Shi W., Li S., Pan D.Z. (2020). A 13.5-ENOB, 107-μW Noise-Shaping SAR ADC With PVT-Robust Closed-Loop Dynamic Amplifier. IEEE J. Solid-State Circuits.

[10] Straayer M.Z., Perrott M.H. (2009). A Multi-Path Gated Ring Oscillator TDC With First-Order Noise Shaping. IEEE J. Solid-State Circuits.

[11] McNeill J., Coln M.C.W., Larivee B.J. (2005). “Split ADC” Architecture for Deterministic Digital Background Calibration of a 16-bit 1-MS/s ADC. IEEE J. Solid-State Circuits.

[12] Ghosh A., Pamarti S. (2015). Linearization Through Dithering: A 50 MHz Bandwidth, 10-b ENOB, 8.2 mW VCO-Based ADC. IEEE J. Solid-State Circuits.

[13] Elkholy A., Anand T., Choi W.-S., Elshazly A., Hanumolu P.K. (2015). A 3.7 mW Low-Noise Wide-Bandwidth 4.5 GHz Digital Fractional-N PLL Using Time Amplifier-Based TDC. IEEE J. Solid-State Circuits.

[14] Ohhata K. (2019). A 2.3-mW, 1-GHz, 8-Bit Fully Time-Based Two-Step ADC Using a High-Linearity Dynamic VTC. IEEE J. Solid-State Circuits.

[15] Ramkaj A.T., Strackx M., Steyaert M.S.J., Tavernier F. (2018). A 1.25-GS/s 7-b SAR ADC With 36.4-dB SNDR at 5 GHz Using Switch-Bootstrapping, USPC DAC and Triple-Tail Comparator in 28-nm CMOS. IEEE J. Solid-State Circuits.

[16] Babayan-Mashhadi S., Lotfi R. (2014). Analysis and Design of a Low-Voltage Low-Power Double-Tail Comparator. IEEE Trans. Very Large Scale Integr. (VLSI) Syst..

[17] Zhang W., Zhang M., Zhu Y., Martins R.P., Chan C.-H. A PVT-Robust 8b 20GS/s Time-Interleaved SAR ADC with Quantization-Embedded Current-Mode Buffer and Differ-Based Dither Timing Skew Calibration. Proceedings of the 2024 IEEE Custom Integrated Circuits Conference (CICC).

[18] Ali A.M., Dinc H., Bhoraskar P., Bardsley S., Dillon C., McShea M., Periathambi J.P., Puckett S. (2020). A 12-b 18-GS/s RF Sampling ADC With an Integrated Wideband Track-and-Hold Amplifier and Background Calibration. IEEE J. Solid-State Circuits.

[19] Ragab K., Sun N. A 12b ENOB, 2.5MHz-BW, 4.8mW VCO-based 0–1 MASH ADC with Direct Digital Background Nonlinearity Calibration. Proceedings of the 2015 IEEE Custom Integrated Circuits Conference (CICC).

[20] Gu M., Zhong Y., Jie L., Sun N. A 12b 3GS/s Pipelined ADC with Gated-LMS-Based Piecewise-Linear Nonlinearity Calibration. Proceedings of the 2025 IEEE International Solid-State Circuits Conference (ISSCC).

[21] Miyazaki D., Yagishita Y., Takasaki M., Nagata S., Nogamida T., Abe Y., Tomita K., Hasebe K., Kikkawa T., Yoshizawa S. An 8b 20GS/s Time-Interleaved ADC with 2.6mW 1GS/s Hybrid Voltage/Time-Domain Sub-ADC in 12nm FinFET. Proceedings of the IEEE International Solid-State Circuits Conference (ISSCC).

[22] Zhu S., Wu B., Cai Y., Chiu Y. (2018). A 2-GS/s 8-bit Non-Interleaved Time-Domain Flash ADC Based on Remainder Number System in 65-nm CMOS. IEEE J. Solid-State Circuits.

[23] Ohhata K., Hayakawa D., Sewaki K., Imayanagida K., Ueno K., Sonoda Y., Muroya K. (2018). A 900-MHz, 3.5-mW, 8-bit Pipelined Subranging ADC Combining Flash ADC and TDC. IEEE Trans. Very Large Scale Integr. (VLSI) Syst..

[24] Oh D.-R., Moon K.-J., Lim W.-M., Kim Y.-D., An E.-J., Ryu S.-T. An 8b 1GS/s 2.55mW SAR-Flash ADC with Complementary Dynamic Amplifiers. Proceedings of the IEEE Symposium on VLSI Circuits.

[25] Zhang C., Wei J., Chen Y., Liu M., Yang Y. (2023). A 0.004-mm^2^ 3.65-mW 7-Bit 2-GS/s Single-Channel GRO-Based Time-Domain ADC Incorporating Dead-Zone Elimination and On-Chip Folding-Offset Calibration in 28-nm CMOS. IEEE J. Solid-State Circuits.

